# De Novo Transcriptome Assembly and Annotation of Liver and Brain Tissues of Common Brushtail Possums (*Trichosurus vulpecula*) in New Zealand: Transcriptome Diversity after Decades of Population Control

**DOI:** 10.3390/genes11040436

**Published:** 2020-04-17

**Authors:** Arsalan Emami-Khoyi, Shilpa Pradeep Parbhu, James G. Ross, Elaine C. Murphy, Jennifer Bothwell, Daniela M. Monsanto, Bettine Jansen van Vuuren, Peter R. Teske, Adrian M. Paterson

**Affiliations:** 1 Center for Ecological Genomics and Wildlife Conservation, University of Johannesburg, Auckland Park 2006, South Africa; ekarsalan@gmail.com (A.E.-K.); shilpa.parbhu@yahoo.com (S.P.P.); dmonsanto119@gmail.com (D.M.M.); bettinevv@uj.ac.za (B.J.v.V.); pteske101@gmail.com (P.R.T.); 2 Department of Pest-management and Conservation, Faculty of Agriculture and Life Sciences, Lincoln University, Lincoln 7647, New Zealand; james.ross@lincoln.ac.nz (J.G.R.); ecmurphy31@gmail.com (E.C.M.); Elaine.Murphy@lincoln.ac.nz (J.B.)

**Keywords:** *De novo* transcriptome assembly, common brushtail possum, liver, brain cerebral cortex, drug and xenobiotic metabolism, chemical toxicants

## Abstract

The common brushtail possum (*Trichosurus vulpecula*), introduced from Australia in the mid-nineteenth century, is an invasive species in New Zealand where it is widespread and forms the largest self-sustained reservoir of bovine tuberculosis (*Mycobacterium bovis*) among wild populations. Conservation and agricultural authorities regularly apply a series of population control measures to suppress brushtail possum populations. The evolutionary consequence of more than half a century of intensive population control operations on the species’ genomic diversity and population structure is hindered by a paucity of available genomic resources. This study is the first to characterise the functional content and diversity of brushtail possum liver and brain cerebral cortex transcriptomes. Raw sequences from hepatic cells and cerebral cortex were assembled into 58,001 and 64,735 transcripts respectively. Functional annotation and polymorphism assignment of the assembled transcripts demonstrated a considerable level of variation in the core metabolic pathways that represent potential targets for selection pressure exerted by chemical toxicants. This study suggests that the brushtail possum population in New Zealand harbours considerable variation in metabolic pathways that could potentially promote the development of tolerance against chemical toxicants.

## 1. Introduction

New Zealand separated from other landmasses approximately 85 million years ago and has remained largely isolated ever since [1]. Ecological isolation created distinctive evolutionary dynamics in the archipelago rarely seen elsewhere on the planet. By the time the first humans colonised in approximately 1300 AD [2,3], no native land mammals, other than three species of bats, were present [4]. The negative impacts of two consecutive waves of human colonisation triggered a series of fundamental alterations in the biological diversity of New Zealand. The introduction of three species of rats (*Rattus* spp.), three species of mustelids (*Mustela* spp.), and the common brushtail possum (*Trichosurus vulpecula*), in combination with extensive human hunting and habitat destruction, triggered a spiral of catastrophic events that drove fifty-nine bird species extinct and severely threatens the survival of other native species, including endemic marine mammals, native bat species, frogs, and much of the invertebrate fauna [5,6].

The common brushtail possum (hereafter ‘brushtail possum’) was introduced into New Zealand from localities in Tasmania and the eastern Australian mainland in the 1850s to establish a profitable fur industry [7,8]. Soon after its introduction, the species established itself in the wild, proliferated, and spread across all habitats in the archipelago. In these new habitats, brushtail possums reached population densities far exceeding those in their native Australian range [4]. The phenomenal success of brushtail possums in New Zealand has been linked to the high abundance of palatable and nutritious food sources, and the presence of fewer parasites, predators, and competitors [9]. The scale of devastation upon New Zealand’s endemic ecosystems prompted the brushtail possum’s designation as both an agricultural pest and a major threat to New Zealand’s unique flora and fauna [9,10]. Wild populations of brushtail possums also harbour a self-sustained reservoir of bovine tuberculosis (*Mycobacterium bovis*) that threatens New Zealand’s dairy and livestock industries [4]. As a result, the brushtail possum has been subject to decades of targeted population control operations.

Conservation and agricultural authorities in New Zealand regularly apply a series of population eradication and control measures to mitigate the negative impacts of brushtail possums on native ecosystems. Since 1957, aerial dispersal of cereal baits containing the chemical toxicant sodium fluoroacetate (or ‘compound 1080′) has constituted the cornerstone of management plans to control brushtail possums and other invasive mammals in New Zealand [10,11]. Synthetic compound 1080 is a water-soluble salt modelled on the naturally occurring plant toxin fluoroacetate [12]. Several plant species across the globe, such as *Gastrolobium* spp. and *Oxyylobium* spp., use this toxicant as a deterrent against herbivory, including species within the possum’s native Australian home range [13,14,15].

Brushtail possum populations in New Zealand face a combination of stressors and human-induced selection pressures that differ from those in their native Australia. The speed of local adaptation typically found in many invasive species suggests that the genetic recipe for adaptation predominately arises from standing genetic variations present in the founder populations [16,17]. Triggs and Green [18] and Sarre et al. [19] reported that current populations of the brushtail possum in New Zealand originated from two distinct subspecies, *Trichosurus vulpecula fuliginosus* from Tasmania and *T. vulpecula vulpecula* from mainland Australia, and that extensive hybridisation took place following their introduction to New Zealand. Genetic variations among the founder populations propagated in the non-native range and most likely became reshuffled into new genotypes that do not occur in Australia.

Recent advances in DNA sequencing technology and high-performance computing have facilitated the comprehensive study of functional regions in eukaryote genomes. Variability in the genome has made it possible to address some fundamental questions in evolutionary genetics, such as the prediction of pathways associated with fruit colour [20], intraspecific variation in morphology and behaviour [21], susceptibility to pathogens [22,23,24,25], responses to chemicals [23], adaptation to environmental changes [26], species diagnostics markers in endangered shrimps [27], sex determination [28], and changes in the expression profiles of an organ during embryonic development [29].

Understanding the brushtail possum’s evolutionary dynamics in New Zealand is hindered by a paucity of available genomic resources for this species [30]. In particular, the evolutionary consequences of more than half a century of intensive population control measures on its genetic diversity and population structure merit in-depth investigation. To address these fundamental questions, genomic resources are required.

In the current study, we characterise the functional content and genetic diversity of brushtail possum liver and brain cerebral cortex transcriptomes, tissues of major importance in responding to chemical toxicants and in modulating complex behaviour [31,32,33,34]. The core metabolic pathways in these tissue types are among the primary targets of selective pressures imposed by anthropogenic chemicals [35,36,37]. This study serves as a stepping-stone for a better understanding of the brushtail possum’s evolutionary dynamics in New Zealand and will contribute towards the optimisation of a comprehensive population management plan for the species, in line with the New Zealand Predator-Free 2050 horizon objectives.

## 2. Materials and Methods

### 2.1. Animal Ethics Statement

Animal ethics approval for this study was obtained from the Lincoln University Animal Ethics Committee (AEC 586). Approved standard operation procedures were used for both animal husbandry and euthanasia. All necessary steps were taken to minimise the pain and suffering of the subject animals.

### 2.2. Specimen Collection

In total, four brushtail possums (two males and two females) were live trapped on the east and west coasts of New Zealand’s two main islands (Banks Peninsula and Buller, South Island, and Taranaki and Hawkes Bay, North Island). The animals were transferred to the wild animal husbandry facilities (Johnstone Memorial Laboratory) at Lincoln University. On the day of specimen collection, sex, weight, and health status of each animal were recorded by Lincoln University’s wildlife animal facility staff. The brushtail possums were humanely euthanised by intracardiac administration of 300 mg of Pentobarbital. The dissection and collection of the liver and cerebral cortex biopsies were performed after the death of the animals was confirmed on site. Approximately 1 *mm*^3^ tissue from liver and brain cerebral cortex were excised, transferred to 10:1 volume of RNAlater solution (Qiagen-Hilden) and stored at −80 °C until RNA was extracted within one week after specimen collection. Liver cells are assumed to be more homogeneous in their gene expression profiles, but see Reference [38], whereas brain cell expression profiles often differ considerably between different brain regions [39,40]. To account for variations in the gene expression profiles in the possums’ cerebral cortex, brain biopsies from three individuals were excised from different sections of the proximal cerebral cortex in the left and right cortical lobes. The remaining biopsy was taken from deeper white matter in the cerebral cortex. A wildlife histologist examined all brain biopsies to validate their origin.

### 2.3. Nucleic Acid Extraction, Genomic Library Preparation, and Sequencing

Total RNA was purified from liver and brain tissue using Qiagen’s RNeasy Mini Kit (Hilden, Germany). The extracted RNA was eluted in 50–100 µl of water. The quality of the purified RNA was measured using Agilent’s Bioanalyzer (Santa Clara, United States) to obtain an RNA Integration Number score before proceeding with complementary DNA preparation. Nugen’s Ovation RNA-Sequencing System (Redwood City, United States), automated on an Apollo324 liquid handler (Takara Bio, Kyoto, Japan), was used to create complementary DNA using a single primer isothermal amplification method and 100 ng of template RNA. The resulting complementary DNA was quantified on a Nanodrop spectrophotometer (ThermoFisher Scientific, Massachusetts, United States) and sheared to 200 bp fragments using the Covaris M220 ultrasonicator (Woburn, Unites States). Illumina-compatible sequencing libraries were prepared using KAPA Biosystem’s LTP library preparation kit (Pleasanton, United States). Individual sequencing adapters with sample-specific barcodes (Bioo Scientific, Austin, United States) were ligated to the end-repaired fragments, the resulting libraries were cleaned using AMPure beads (Beckman-Coulter, Brea, United States) and amplified for ten PCR cycles with KAPA’s HiFi polymerase. The concentration of each library was quantified separately using qPCR on the Quantstudio 5 (Applied Biosystems, Foster City, United States) and the fragment sizes were verified using the Agilent Bioanalyzer. All sequencing libraries were normalised to 2 nM concentration before pooling for multiplex sequencing on two 1 × 50 flow cells on an Illumina HiSeq2000 platform (San Diego, United States) at the Arizona State University genomics core facility.

### 2.4. Transcriptome Assembly, Variant Calling and Functional Annotation

Leading and trailing low-quality or N bases (Phred quality below three), all base pairs with average Phred score < 20, and Illumina adapter contaminations were removed using Trimmomatic v0.39 [41]. A reference transcriptome for each tissue type was assembled de novo in Trinity v2.8.6 (Inchworm, Chrysalis, and Butterfly modules) [42] using default settings. Resulted transcripts were clustered into unique clusters by utilising cd-hit-est from the CD-HIT v4.7 package and setting identity parameters to 98% [43]. The longest reading frame within each transcript was predicted in TransDecoder v5.5.0 [44]. All transcripts were functionally annotated using the Trinotate pipeline [45]. In Trinotate, all transcripts were searched against known proteins and core metabolic pathways deposited in Swiss-Prot protein (https://www.uniprot.org/), NCBI non-redundant protein (https://www.ncbi.nlm.nih.gov/refseq/), and Kyoto Encyclopaedia of Genes and Genomes (KEGG) (https://www.genome.jp/kegg/) databases. In addition, the protein domains for all transcripts were predicted in HMMER v3.1 [46] based on the similarity to the known proteins in the Pfam (https://pfam.xfam.org/) database. Transmembrane proteins and ribosomal RNA were separately identified using TmHMM v2 [47] and RNAMMER v1.2 [48], respectively. The Benchmarking Universal Single-Copy Orthologs (BUSCO V3) pipeline [49] was executed in protein mode to evaluate the completeness of the gene content of the reference transcriptome of each tissue compared to the core mammalian gene content (Mammalia-0db-10, created on 2019-11-20).

From all specimens, quality-filtered sequences were aligned against the corresponding assembled transcriptome using Bowtie2 [50], BWA-ALN [51], and BWA-MEM aligners [52] and mapping statistics from each alignment file were estimated separately in SAMtools v1.9 flagstat script [53].

A combination of SAMtools v1.9 mpileup command [53] and Varscan2 v2.3.7 [54] were used to identify single nucleotide polymorphic sites (SNPs) in each tissue type. Default settings were used for variant calling, an exception being the *p*-values in Varscan2, which were lowered to 0.001 to increase prediction accuracy. Functional impacts of the polymorphic sites on protein sequences were predicted using the KisSplice2reftranscriptome v1.3.3 pipeline [55].

Two datasets were created and investigated in parallel to unravel variability in the functional content of each tissue type. The first consisted of ten transcripts within each tissue’s reference transcriptome that showed the highest number of polymorphic sites, as reported in the Varscan2 output files. In the second dataset, the abundance of each transcript was estimated in RSEM v1.3.1 [56], and 5% of the transcripts with the highest normalised level of expression (estimated as per million mapped reads, TPM) were sub-selected for further functional analysis.

The enrichments of the ten most polymorphic transcripts in each tissue type, for particular biological functions in terms of Gene Ontology categories (GO terms) [57], were investigated using the g: Profiler online server [58] and the annotated genome of the grey short-tailed opossum, *Monodelphis domestica*, was used as the closest high-quality annotated gene set.

The tissue-specific expression profiles and intra-individual variations in the functional content of polymorphic sites were statistically tested using the WEGO v2.0 online tool [59] and visualised using a combination of the REVIGO online server [60] and CirGO [61]. The signal of pervasive selection (the ratio of non-synonymous to synonymous amino acid substitution, dN/dS ratio) in one particular liver transcript with an exceptionally high number of polymorphic sites was tested using a fixed-effects likelihood model [62] implemented in the online server Datamonkey [63].

The functional content of the assembled transcripts and two generated datasets, in terms of core metabolic pathways, was reconstructed by mapping transcripts against the KEGG database using the KEGG mapper online server [64] and results were summarized in GAEV [65].

## 3. Results

The histological reports confirmed that three of the brain cerebral cortex biopsies originated from the proximal cortex, while the remainder originated from deeper within the cerebral cortex white matter (Supplementary Materials Figures S1–S4).

The RNA sequencing run yielded 183,517,606 and 178,725,338 raw sequences from liver and brain cerebral cortex, respectively. Liver tissue sequences were assembled into 58,001 transcripts (candidate genes and isoforms), with a mean contig length of 813.37 and a GC content of 42.57%. From the cerebral cortex brain tissue, Trinity assembled sequences with a total of 64,735 transcripts, a mean contig length of 738.13, and a GC content of 42.68% (Table 1). CDHIT subsumed all transcripts into 57,221 and 63,679 unique clusters with more than 98% sequence identity for liver and brain cerebral cortex, respectively.

The semi-global BWA-MEM aligner performed better, in terms of mapping quality and percentage of aligned sequences, compared to two other aligners, and was selected for downstream analysis. The overall alignment rate was estimated at 90.93% for liver sequences and 79.80% for brain cerebral cortex sequences. The alignment rate in both tissue types lies within the recommended range in the Trinity pipeline for a complete reference transcriptome assembly (https://github.com/trinityrnaseq/trinityrnaseq/wiki/RNA-Seq-Read-Representation-by-Trinity-Assembly). From a total of 9226 searched BUSCOs, 3290 and 2446 of the core mammalian’s complete BUSCOs were identified in the hepatic and brain cerebral cortex transcriptomes, respectively (Supplementary Materials Table S1). Tissue-specific transcriptomes, especially in non-model organisms, are very unlikely to produce complete mammalian BUSCOs and that should be interpreted within the biological context of the study, rather than as a direct metric for estimating the completeness of the assembled transcriptomes.

Varscan2 identified 35,300 (one SNP for every 1339 bp) and 23,805 (one SNP for every 2007 bp) variable sites in liver and brain cerebral cortex transcripts, respectively. The number of polymorphic sites with transitions was estimated to be three times higher than the number of transversions in both tissue types. The polymorphism landscape in brushtail possum cerebral cortex was more heterogeneous compared to hepatic cells. Only 25% (5939 out of 23,805) of the polymorphic sites in cerebral cortex tissues were successfully genotyped across all four sampled tissues compared to 70% shared polymorphisms (24,924 out of 35,225) among hepatic tissues.

KisSplice identified 34,329 (22,887 synonymous and 11,442 synonymous) and 4695 (3244 synonymous and 1451 non-synonymous) codon substitutions in hepatic and cerebral cortex tissues, respectively. The reported high levels of heterogeneity in gene expression profiles among different sections of the cerebral cortex provide the most likely explanation for the observed pattern of codon substitutions.

In the hepatic cells, non-synonymous mutations of the codons GTT to ATT, ACG to ATG (substitution of amino acid valine to isoleucine) and GTC to ATC (substitution of amino acid threonine to methionine) had the highest frequency. In the brain cerebral cortex, non-synonymous mutation of the codons GTG to CTG (substitution of amino acid valine to leucine) AAA to AGA (substitution of amino acid lysin to arginine) and AGT to AAT (substitution of amino acid serine to asparagine) were prevalent. The highest rate of synonymous amino acid substitutions was observed for aspartate (mutation in codon GAC to GAT or GAT to GAC) and asparagine (mutations in the codon AAC to AAT) in the hepatic cells and for amino acids aspartate (mutation in the codon GAC to GAT) and proline (mutation in the codon CCA to CCG) in the brain cortical cells (Supplementary Materials Tables S4 and S5)

A transcript in the liver was of particular interest because of an exceptionally large number of polymorphic sites (127 SNPs). The BLASTX search of this transcript showed high sequence identity (88% similarity) to various isoforms of multidrug resistance proteins reported in koala, *Phascolarctos cinereus* (NCBI reference XP_020843128.1), and common wombat, *Vombatus ursinus* (NCBI Reference Sequence: XP_027703707.1). In all four brushtail possums, alignment of the protein-coding sequences of this transcript demonstrated 19 candidate sites that were under pervasive purifying selection (dN/dS < 1, *p*-value < 0.05). The majority of the non-synonymous amino acid substitutions in these transcripts (6 out of 30) were in the form of valine to isoleucine substitutions. The synonymous substitution for amino acids alanine and isoleucine (11 out of 45) constituted a considerable number of substitution patterns in the same transcript.

Searching all assembled transcripts against multiple databases showed a considerable number of uncharacterised transcripts within both tissue types (Supplementary Materials Figures S5 and S6).

**Figure 1:**
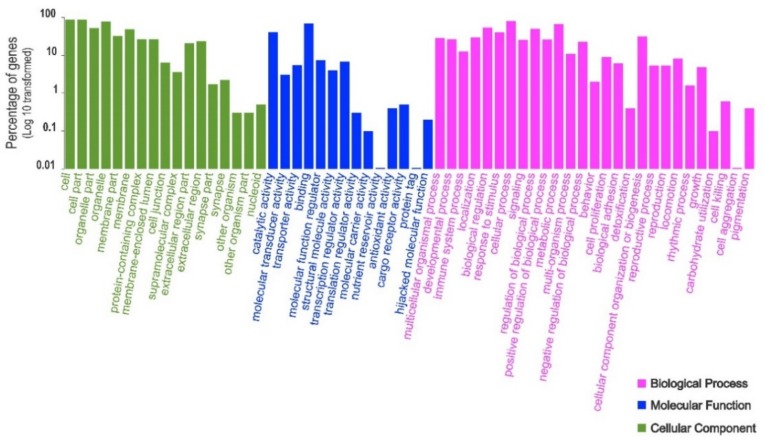
Functional annotation of all polymorphic transcripts in brushtail possum liver cells based on Gene Ontology (GO) categorisation. The X-axis indicates GO functions and the Y-axis shows the percentage of transcripts in log (10) scales.

**Figure 2:**
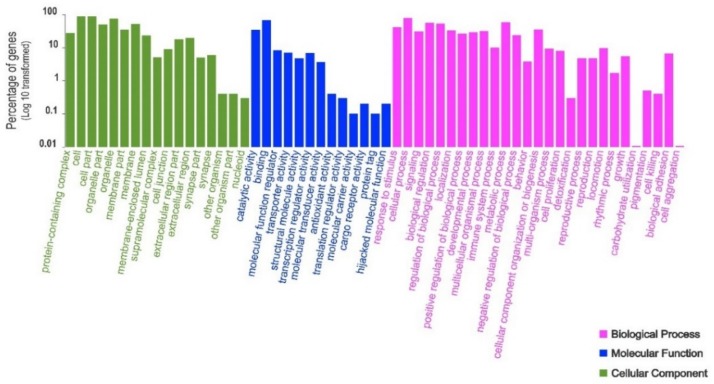
Functional annotation of all polymorphic transcripts in brushtail possum brain cerebral cortex cells based on Gene Ontology (GO) categorisation. The X-axis indicates GO functions and the Y-axis shows the percentage of transcripts in log (10) scales.

The analysis of the functional content of both transcriptomes in terms of Gene Ontology (GO) illustrated that GO terms associated with biochemical pathways involved in cellular components (cells, organelle and organelle parts, membrane, and extracellular regions), molecular functions (catalytic activity, binding, molecular function regulators, and molecular transducer activity), and biological processes (response to stimulus, cellular process, biological regulation, metabolic process, and biogenesis) dominated the functional diversity of both tissue types ( Figure 1; Figure 2).

**Figure 3:**
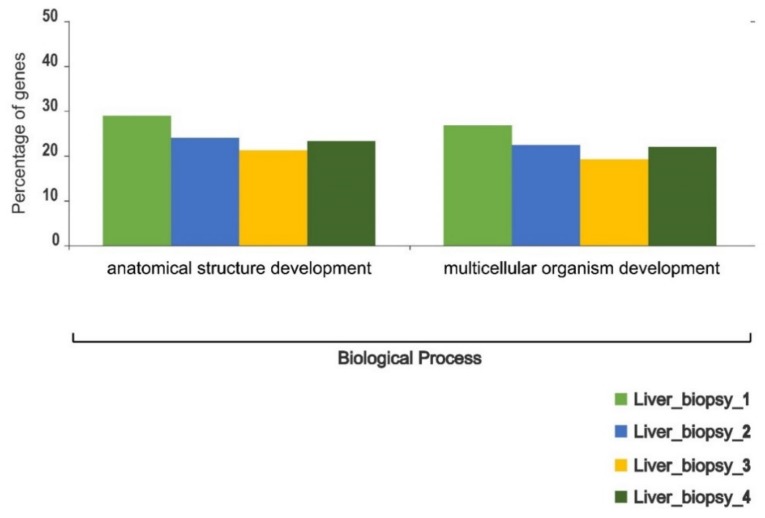
Intra-individual variation in the functional content of polymorphic transcripts in brushtail possum liver cells based on Gene Ontology (GO) categorisation. The X-axis indicates GO functions and the Y-axis shows the percentage of transcripts.

**Figure 4:**
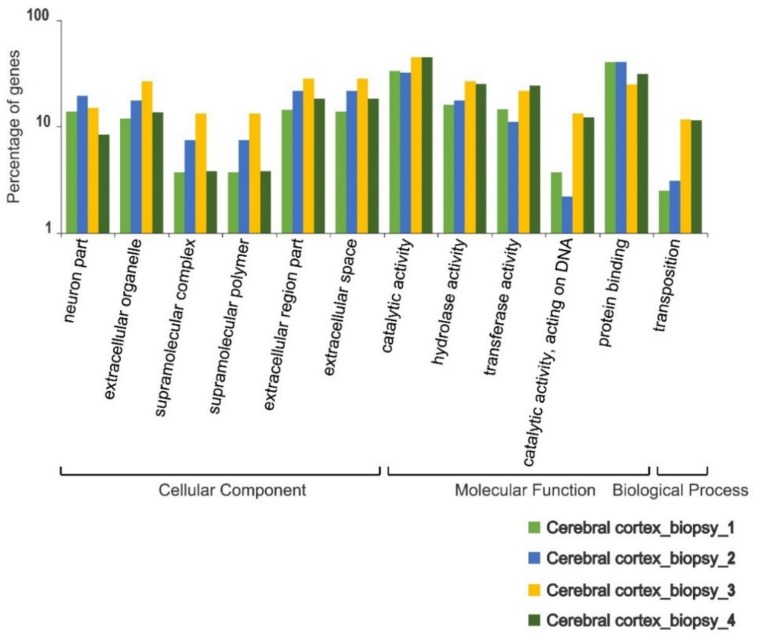
Intra-individual variation in the functional content of polymorphic transcripts in brushtail possum cerebral cortex cells based on Gene Ontology (GO) categorisation. The X-axis indicates GO functions and the Y-axis shows the percentage of transcripts.

The hepatic cells were more homogeneous in their gene ontology profiles across individuals. Only two metabolic pathways involved in the biological process, anatomical structure developments and multicellular organism developments, showed statistically significant differences in the number of associated polymorphic sites (^2^ test *p*-value < 0.05) across the four individuals (Figure 3). The brain biopsies taken from different sections of the cerebral cortex demonstrated more heterogenous profiles in their polymorphism landscape. Twelve metabolic pathways involved in cellular components (neurone part, extracellular organelle-complex, and extracellular space), molecular functions (catalytic, hydrolase, transferase, and protein-binding activities), and a biological process linked to transposition showed a significant level of variation across different parts of the cerebral cortex (^2^ test *p*-value < 0.05) (Figure 4).

**Figure 5:**
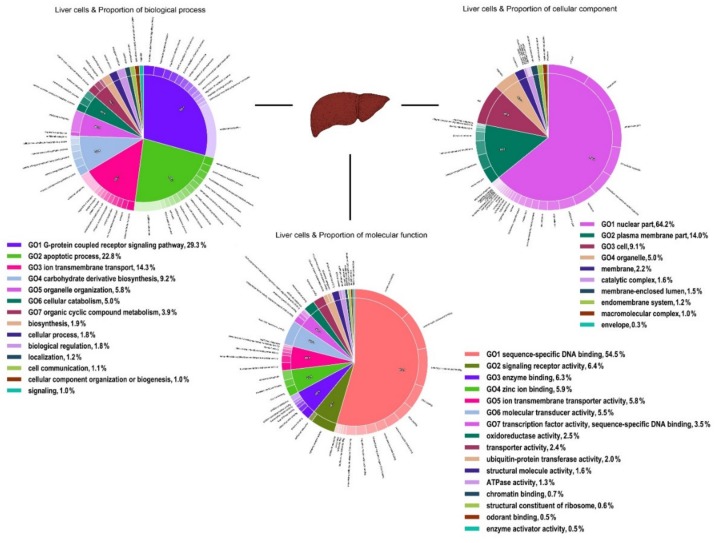
Functional enrichment of 10 transcripts with the highest levels of polymorphism, based on Gene Ontology (GO) categorisation, in brushtail possum liver cells compared to the grey short-tailed opossum (*Monodelphis domestica*) complete gene set.

**Figure 6:**
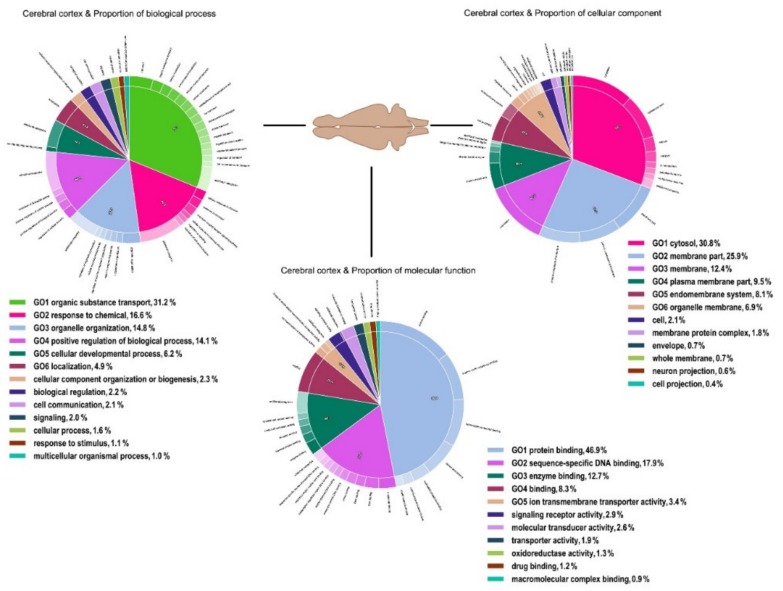
Functional enrichment of 10 transcripts with the highest levels of polymorphism, based on Gene Ontology (GO) categorisation, in brushtail possum cerebral cortex cells compared to the grey short-tailed opossum (*Monodelphis domestica*) complete gene set.

Comparing the liver and brain datasets consisting of the ten most polymorphic sites against the closely related grey short-tailed opossum illustrated significant enrichment for various metabolic pathways (Figure 5; Figure 6).

The dataset of 5% highly expressed transcripts illustrated that the expression profile between two tissue types differs significantly (^2^ test *p*-value < 0.05) in the metabolic pathways linked to 58 GO terms (Supplementary Materials Figure S7).

The metabolic pathways analysis of the transcripts against the KEGG database indicated that the liver transcriptome was linked to 394 core metabolic pathways and 23 complete metabolic modules. Brain cerebral cortex transcriptome was involved in 379 core metabolic pathways and eight complete metabolic modules (Supplementary Materials Tables S2 and S3).

In the liver tissue, the most polymorphic transcripts were involved in eight KEGG metabolic pathways including lipid metabolism, nucleotide metabolism, amino acid metabolism, cofactor and vitamin metabolism, xenobiotics biodegradation and metabolism, and the endocrine system. Mapping the ten most polymorphic transcripts in the brain cerebral cortex against the KEGG database indicated their involvement in 14 core biological pathways, including core metabolic pathways, biosynthesis of secondary metabolites, metabolism of cofactors and vitamins, signal transduction, signalling molecules and interaction, transport and catabolism, the cellular community, and the excretory system.

The dataset consisting of 5% of the most abundant transcripts of hepatic cells was linked to 354 biological pathways and eight complete metabolic modules (Table 2). The highly expressed transcripts in the brain cerebral cortex were associated with 342 biological pathways and four complete metabolic modules (Table 3). A total of 22 genes in the liver transcriptomes and ninegenes in the cerebral cortex were directly linked to the metabolic pathways that involved drug and xenobiotic substances metabolism (Table 4; Table 5).

## 4. Discussion

The potential of pests, pathogens, and invasive species to evade conventional population control measures represents a major threat across the globe [67]. Resistance against antibiotics and anthropogenic toxicants occurs in all domains of life, from bacteria to arthropods and vertebrates [68,69,70,71,72]. The genomic basis of developing resistance in non-rodent vertebrates continues to be underrepresented in research and suffers from a lack of functional genomic studies. The current study is the first to characterise the functional content and diversity of the liver and brain cerebral cortex transcriptomes of a non-rodent mammalian pest, the marsupial common brushtail possum, in New Zealand.

The functional content and polymorphism landscape of liver and brain cerebral cortex transcriptomes in brushtail possums are involved in a diverse array of biological processes. The functional content of these tissue types plays a critical part in the detoxification of xenobiotic substances [73,74] and modulation of complex behaviours [75,76] that potentially promote the development of physiological and behavioural tolerance against chemical compounds.

Enzymes produced from cytochrome P450 gene groups (such as CYP1A2, CYP2E1, CYP3A4, CYP3A5, CYP2B6, CYP2C8, and CYP2C19) dominate the biochemical pathways involved in drug and xenobiotic substance metabolism in brushtail possum hepatic cells. Polymorphisms in this group of heme-containing proteins influence the speed and efficiency of chemical compound breakdown in the liver and other organs [77,78]. Similarly, the CYP2C group of genes has been reported to show high levels of expression in liver tissues of the herbivorous koala (*Phascolarctos cinereus*) [79].

The detoxification of the harmful component of compound 1080, fluoroacetate, takes place in the hepatocytes [14,80,81,82]. Polymorphism in the liver transcripts for the metabolic pathways involved in response to drugs and xenobiotic substances is not unexpected. Nonetheless, an exceptionally high number of polymorphic sites associated with these pathways, particularly a contig with high similarity to the multidrug resistance protein family reported from other marsupials, suggest the potential of standing genomic variation within New Zealand populations that may allow alternative responses to population control chemicals.

Multidrug resistance proteins confer increased drug resistance through a decrease in drug accumulations resulting from increased drug efflux [83,84]. Members of this ATP-binding cassette protein super-family function as active transporters of various organic substances, anionic conjugates, and xenobiotic substances, including glutathione, glucuronide, and sulfate conjugates [85,86]. Metabolic pathways that involve glutathione and a unique glutathione transferase are closely linked to the detoxification of compound 1080 and other similar-acting toxicants in hepatic cells [14,80,81,82]. Moreover, the estimated ratio of synonymous to non-synonymous amino acid substitutions in this transcript indicates that parts of the multi-drug resistance metabolic pathways in brushtail possum hepatic cells may be subject to a significant level of purifying selection against less beneficial genotypes. The pre-adaptations of founder populations with natural exposure to this toxicant in Australia, and the fitness implication of the observed evolutionary dynamics, are yet to be fully understood and require comparative functional genomic studies between a larger sample of brushtail possums in their native range in Australia and invaded habitat in New Zealand.

In the current study, several enriched metabolic pathways were identified in the brushtail possum cerebral cortex transcriptome that regulate a wide range of core neurobiological processes, such as organic substance transport, response and regulation of biological processes, cell communications, neurochemical signalling, and locomotion. The biochemical pathways involved in drug and xenobiotic substance metabolism in the cerebral cortex are largely different from those identified in liver cells. The products of several genes in the brushtail possum cerebral cortex (Table 5) are associated with the development of resistance against platinum-based anti-malignancy drugs in humans. The complex dynamics of resistance involve a combination of decreased drug influx, increased drug efflux, intracellular detoxification by glutathione, decreased binding, increased DNA repair, decreased mismatch repair, and defective apoptosis [87,88]. However, the physiological significance of these metabolic pathways in the brushtail possum cerebral cortex in response to the chemicals remains to be investigated.

The delicate interplay between brain cerebral cortex polymorphisms and modulation of complex behaviours is an active area of research in neurobiology [89,90]. While addressing these associations lies beyond the scope of the current study, our results suggest a considerable variation in the transcriptomic repertoire of thebrushtail possum cerebral cortex that is necessary to modulate complex behaviours. Ross et al. [91] suggested that a single exposure to a sub-lethal dose of chemical toxicants in brushtail possums triggers the development of long-lasting bait-shyness behaviours in wild and captive populations. To what extent bait-shyness represents learned behaviour within an individual subsequent to sub-lethal exposure, and its heritability across multiple generations, requires more neurogenomic research.

Instances of developing tolerance against 1080 have already been reported in some wild and laboratory- populations with natural exposure to fluoroacetate [82], including wild populations of European rabbit (*Oryctolagus cuniculus*) in Australia [71], brushtail possums in south-western Australia [92], and laboratory brown rats (*Rattus norvegicus*) [93]. The current study did not aim to identify the development of practical resistance against toxins in New Zealand’s brushtail possum population. Rather, we demonstrated that these populations harbour considerable levels of functional variation in the metabolic pathways that could potentially promote the development of tolerance against chemicals, such as compound 1080 and other similar-acting toxicants. To determine the sensitivity of the current populations to long-term toxin exposure requires formal toxicology trials to measure the lethal concentrations in the laboratory and over time. The power of genetic data to conclusively predict the development of tolerance against toxins remains limited. The small scale of this study only allows for some preliminary conclusions that are consistent with what we would expect to see from a species that has been under intense anthropogenic selection pressures, including both trapping and toxins, for generations.

As conservation efforts to eradicate invasive mammals intensify towards the objectives of the New Zealand Predator-Free 2050 horizon, we strongly advocate combined and multifaceted population control strategies that simultaneously target different aspects of an invasive species’ life history, behaviour, and physiology in time and space, and thus prevent or delay the potential development of resistance against any specific population control measure [67,94]. The ecological and economic impacts of brushtail possums potentially developing tolerance against population control measures necessitate constant monitoring of current populations and their response to management plans.

## Figures and Tables

**Table 1:** Summary statistics of the reference transcriptome assembly of the New Zealand brushtail possum’s liver and brain cerebral cortex tissue as estimated in QUAST v.4.0 [66].

**Assembly Name**	**Liver Cells**	**Brain Cells**
Number of contigs	58,001	64,735
Number of contigs ≥ 1000 bp	13,361	12,385
Number of contigs ≥ 5000 bp	605	612
Number of contigs ≥ 10,000 bp	19	24
Total length bp	47,176,165	47,783,051
Total length ≥ 1000 bp	29,238,918	27,088,346
Total length ≥ 5000 bp	3,994,586	4,080,570
Total length ≥ 10,000 bp	245,362	318,579
Largest contig	18,159	21,422
GC %	42.57	42.68
N50	1447	1247
N75	607	508
L50	8660	9524
L75	21,240	24,749

**Table 2:** Kyoto Encyclopaedia of Genes and Genomes (KEGG) metabolic pathway names and number of instances identified in the top 5% of the highly expressed genes within the brushtail possum liver transcriptome.

Metabolic Pathway	Number
Global and overview maps	591
Carbohydrate metabolism	134
Energy metabolism	65
Lipid metabolism	118
Nucleotide metabolism	12
Amino acid metabolism	152
Metabolism of other amino acids	25
Glycan biosynthesis and metabolism	17
Metabolism of cofactors and vitamins	54
Metabolism of terpenoids and polyketides	10
Biosynthesis of other secondary metabolites	12
Xenobiotics biodegradation and metabolism	60
Transcription	8
Translation	98
Folding, sorting, and degradation	70
Replication and repair	3
Membrane transport	5
Signal transduction	228
Signalling molecules and interaction	35
Transport and catabolism	116
Cell growth and death	60
Cellular community—eukaryotes	54
Cellular community—prokaryotes	2
Cell motility	23
Immune system	181
Endocrine system	185
Circulatory system	24
Digestive system	67
Excretory system	18
Nervous system	81
Sensory system	14
Development and regeneration	21
Aging	23
Environmental adaptation	49

**Table 3:** Kyoto Encyclopaedia of Genes and Genomes (KEGG) metabolic pathway names and number of instances identified in the top 5% of the highly expressed genes within the brushtail possum cerebral cortex transcriptome.

Metabolic Pathway	Number
Global and overview maps	254
Carbohydrate metabolism	62
Energy metabolism	59
Lipid metabolism	30
Nucleotide metabolism	11
Amino acid metabolism	51
Metabolism of other amino acids	6
Glycan biosynthesis and metabolism	10
Metabolism of cofactors and vitamins	9
Metabolism of terpenoids and polyketides	5
Biosynthesis of other secondary metabolites	6
Xenobiotics biodegradation and metabolism	10
Transcription	14
Translation	104
Folding, sorting and degradation	61
Replication and repair	6
Membrane transport	1
Signal transduction	442
Signaling molecules and interaction	39
Transport and catabolism	128
Cell growth and death	100
Cellular community - eukaryotes	79
Cellular community - prokaryotes	2
Cell motility	32
Immune system	184
Endocrine system	256
Circulatory system	46
Digestive system	66
Excretory system	41
Nervous system	196
Sensory system	29
Development and regeneration	44
Aging	26
Environmental adaptation	68

**Table 4:** Name and description of the putative genes involved in drug and xenobiotic substance metabolic pathways in brushtail possum liver cells as predicted using the KEGG database.

Gene Name	Description
*frmA, ADH5, adhC*	S-(hydroxymethyl)glutathione dehydrogenase/alcohol dehydrogenase
*AOX*	aldehyde oxidase
*DPYD*	dihydropyrimidine dehydrogenase (NADP+)
*MAO, aofH*	monoamine oxidase
*FMO*	dimethylaniline monooxygenase (N-oxide forming)
*UGT*	glucuronosyltransferase
*GST, gst*	glutathione S-transferase
*ndk, NME*	nucleoside-diphosphate kinase
*CES1*	carboxylesterase 1
*DPYS, dht, hydA*	dihydropyrimidinase
*dut, DUT*	dUTP pyrophosphatase
*PIK3R1_2_3*	phosphoinositide-3-kinase regulatory subunit α/β/delta
*CES2*	carboxylesterase 2
*CYP1A2*	cytochrome P450 family 1 subfamily A polypeptide 2
*CYP2E1*	cytochrome P450 family 2 subfamily E polypeptide 1
*GSTK1*	glutathione S-transferase kappa 1
*ADH1_7*	alcohol dehydrogenase 1/7
*CYP3A4*	cytochrome P450 family 3 subfamily A polypeptide 4
*CYP3A5*	cytochrome P450 family 3 subfamily A polypeptide 5
*CYP2B6*	cytochrome P450 family 2 subfamily B polypeptide 6
*CYP2C8*	cytochrome P450 family 2 subfamily C polypeptide 8
*CYP2C19*	cytochrome P450 family 2 subfamily C polypeptide 19

**Table 5:** Name and description of the putative genes involved in drug and xenobiotic metabolic pathways in brushtail possum brain cerebral cortex cells as predicted using the KEGG database.

Gene Name	Description
*CBR1*	carbonyl reductase 1
*hprT, hpt, HPRT1*	hypoxanthine phosphoribosyltransferase
*GST, gst*	glutathione S-transferase
*ndk, NME*	nucleoside-diphosphate kinase
*dut, DUT *	dUTP pyrophosphatase
*PIK3R1_2_3*	phosphoinositide-3-kinase regulatory subunit α/β/delta
*TOP2*	DNA topoisomerase II
*ERK, MAPK1_3*	mitogen-activated protein kinase 1/3
*AKT RAC*	serine/threonine-protein kinase

## Supplementary Materials

The following are available online at https://www.mdpi.com/2073-4425/11/4/436/s1, Figures S1–S4: The histological images and reports of the four brain cerebral cortex biopsies, Table S1: The gene content of the liver and brain transcriptomes estimated in the BUSCO v3, Figures S5 and S6: The number of transcripts that was annotated by producing significant matches against different databases, Figure S7: The Gene Ontology terms of the top 5% highly expressed transcripts that differ significantly between the liver and brain cerebral cortex cells. Table S1. The gene content of the brushtail possum’s liver and brain cerebral cortex transcriptomes compared to the core-mammalian BUSCOs. Tables S2 and S3: Definition and number of KEGG metabolic pathways in the liver and brain cerebral cortex transcriptomes. Tables S4 and S5: Liver and brain synonymous/non-synonymous amino acid substitution. The raw sequences generated for this study were submitted to the Sequence Read Archive (SRA) at the NCBI database under Bio Project submission numbers PRJNA623153 and PRJNA623060.
