# Supplementary material for: De Novo Transcriptome Assembly and Annotation of Liver and Brain Tissues of Common Brushtail Possums (Trichosurus vulpecula) in New Zealand: Transcriptome Diversity after Decades of Population Control
Source: Genes (Basel). 2020 Apr 17;11(4):436. doi: 10.3390/genes11040436 (PMC7230921; doi:10.3390/genes11040436)
Supplement: Supplementary file 1 [file genes-11-00436-s001.pdf]

genes

MDPI

# De Novo Transcriptome Assembly and Annotation of Liver and Brain Tissues of Common Brushtail Possums (Trichosurus vulpecula) in New Zealand: Transcriptome Diversity after Decades of Population Control

## Supplementary Materials

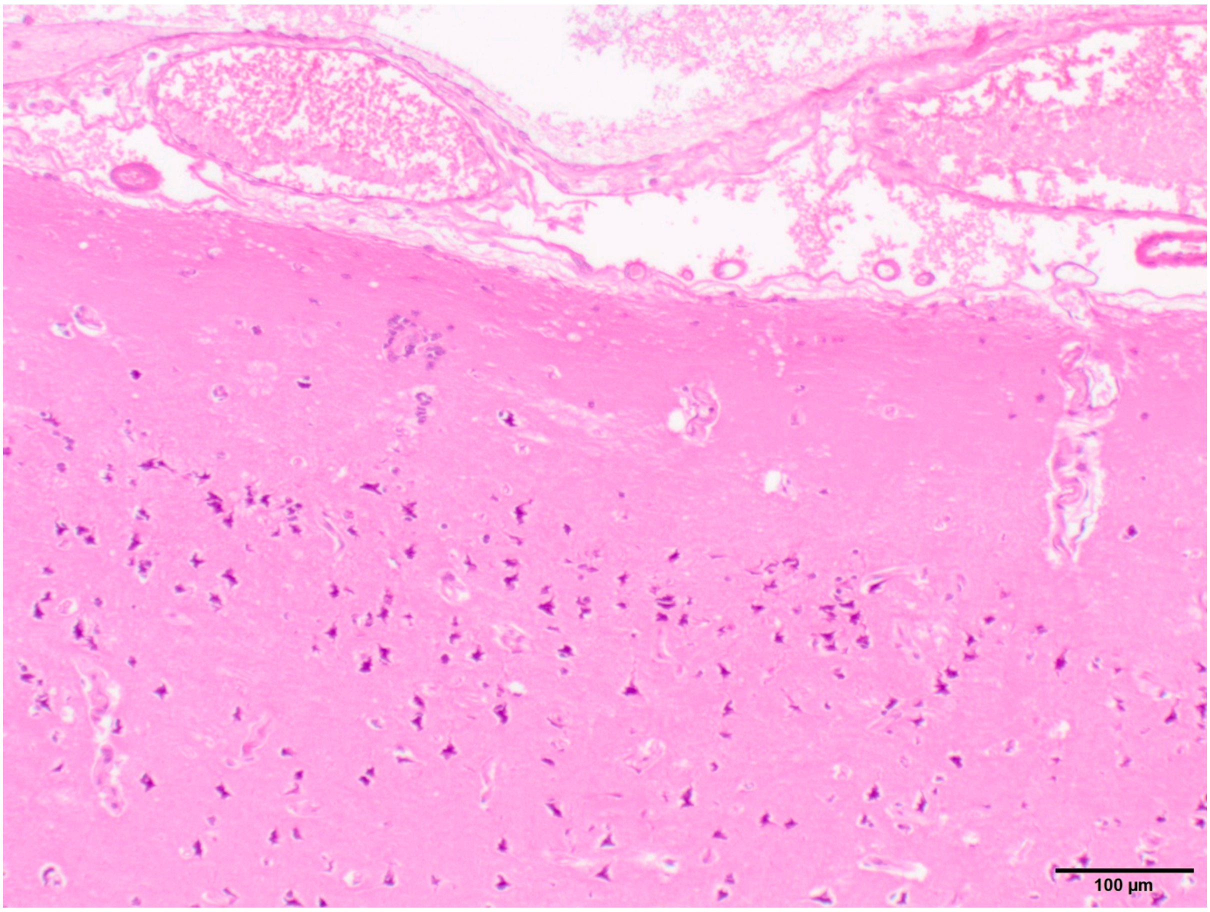


**Figure S1.** Mid magnification image of a brain cerebral cortex from a brushtail possum sample. The image depicts the cerebral cortex using hematoxylin and eosin staining, 100x original magnification, scale bar=100 µm.

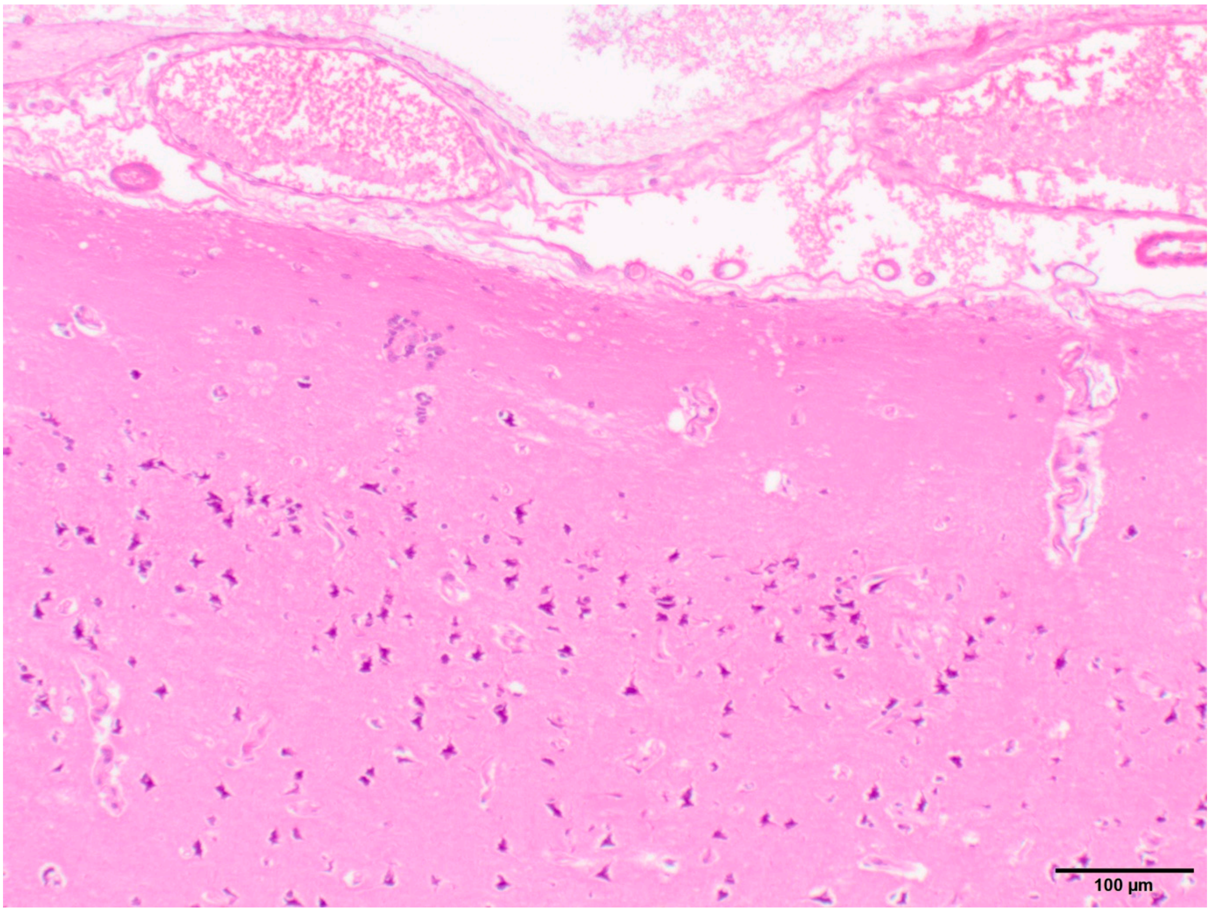


**Figure S2.** Mid magnification image of brain cerebral cortex from a brushtail possum sample. The image depicts the cerebral cortex using hematoxylin and eosin staining, 100x original magnification, scale bar=100 µm.

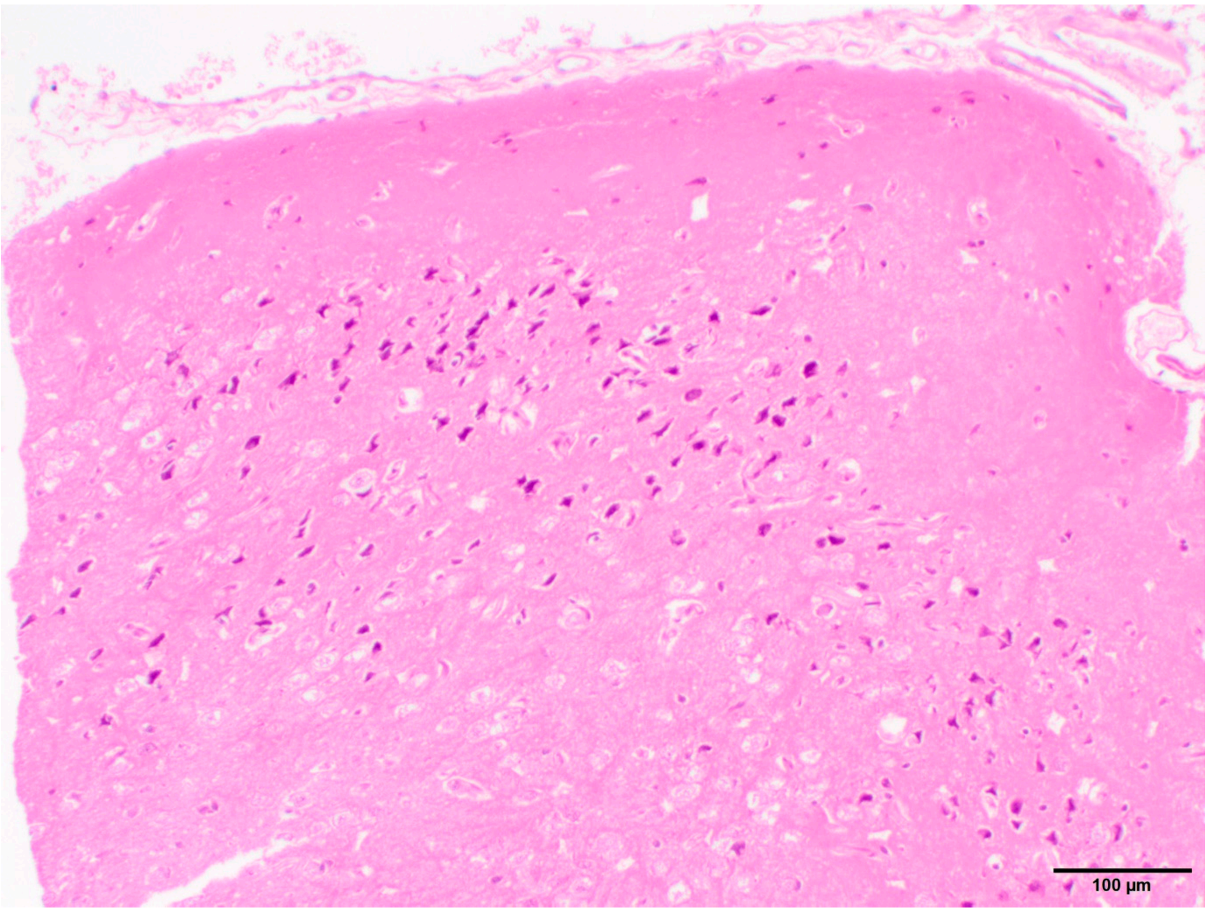


**Figure S3.** Mid magnification image of brain cerebral cortex from a brushtail possum sample. The image depicts the cerebral cortex using hematoxylin and eosin staining, 100x original magnification, scale bar=100 µm.

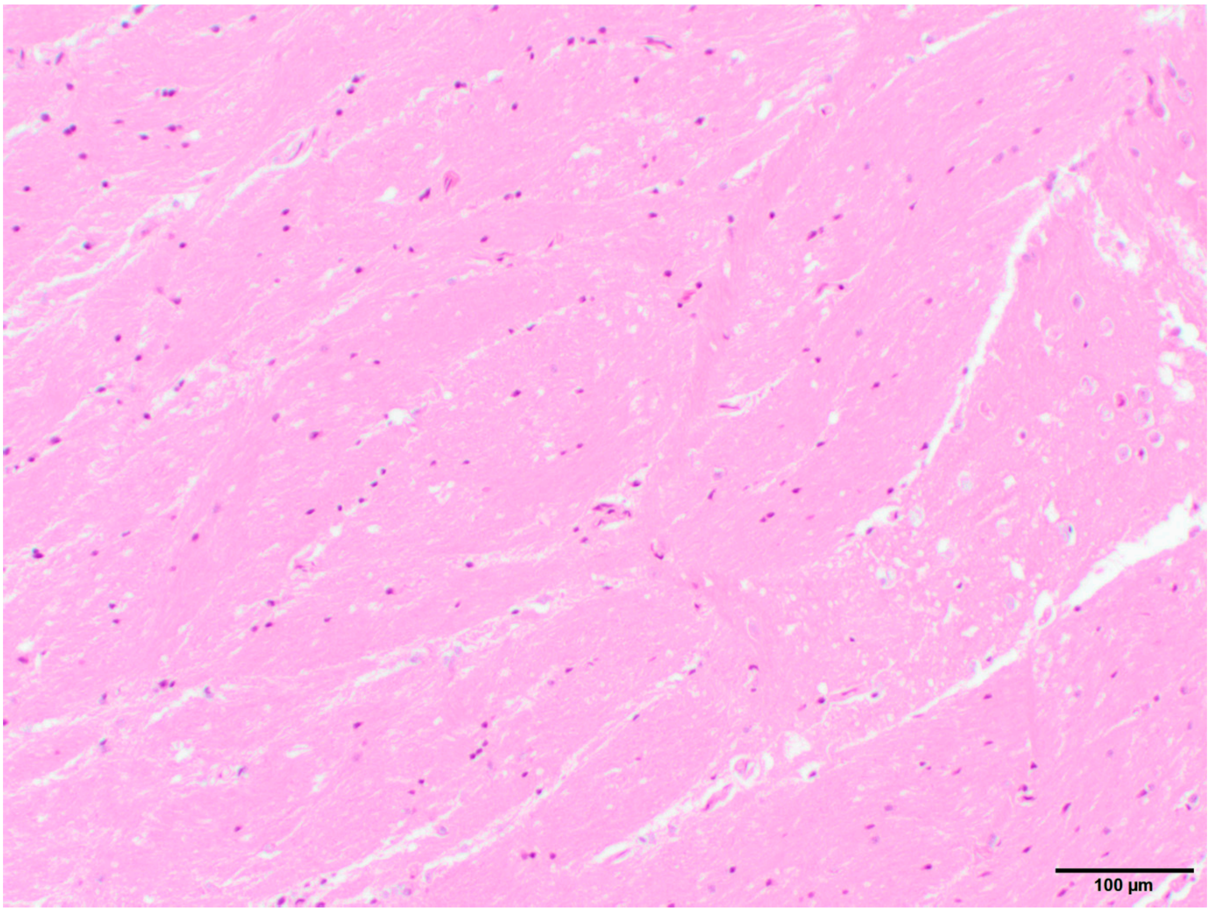


**Figure S4.** Mid magnification image of brain cerebral cortex from brushtail possum sample. The image depicts white matter with scattered glial cells using hematoxylin and eosin staining, 100x original magnification, scale bar=100 µm.

**Table S1.** The gene content of the brushtail possum's liver and brain cerebral cortex transcriptomes compared to the core-mammalian BUSCOs.

| Transcriptome Name | Liver | Brain |
|---|---|---|
| Complete BUSCOs | 3290 | 2446 |
| Complete and single-copy BUSCOs | 2898 | 2163 |
| Complete and duplicated BUSCOs | 392 | 283 |
| Fragmented BUSCOs | 844 | 988 |
| Missing BUSCOs | 5092 | 5792 |
| Total BUSCO groups searched | 9226 | 9226 |

Complete—contig lengths matched to the BUSCO profile; duplicated—contigs found more than once in orthology annotation; fragmented—partially covered; missing—no matches that passed orthology classification tests.

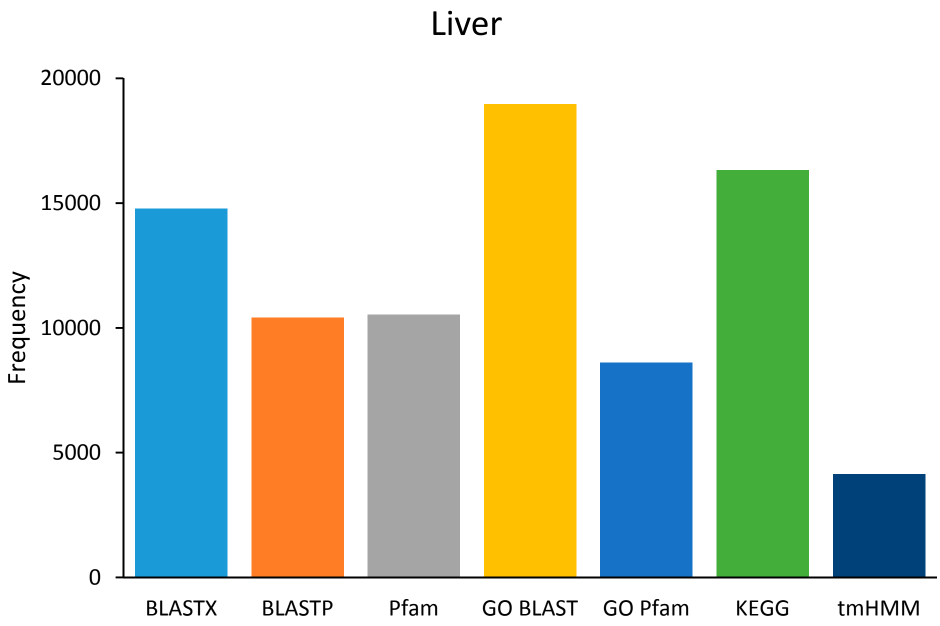


**Figure S5.** Number of liver transcripts that returned significant matches in different databases.

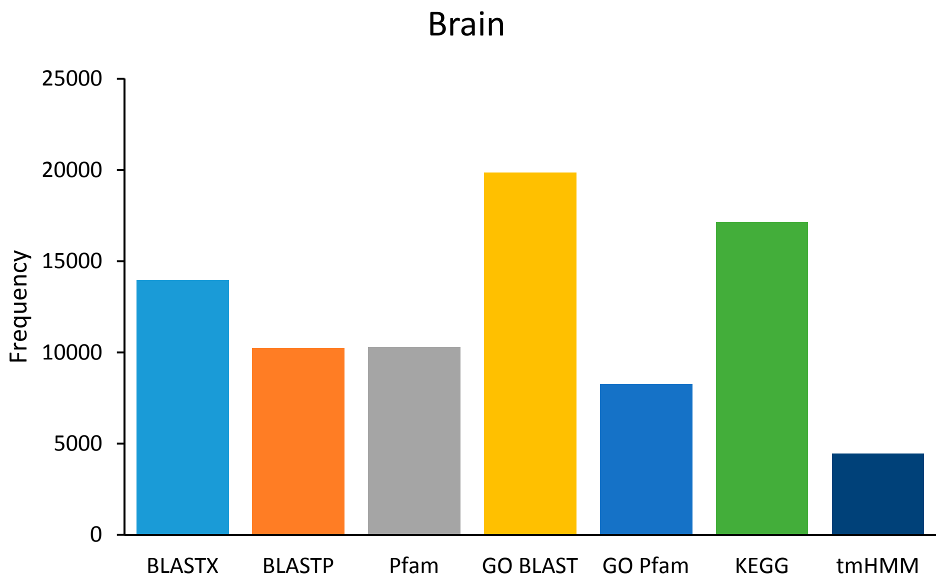


**Figure S6.** Number of brain cerebral cortex transcripts that returned significant matches in different databases.

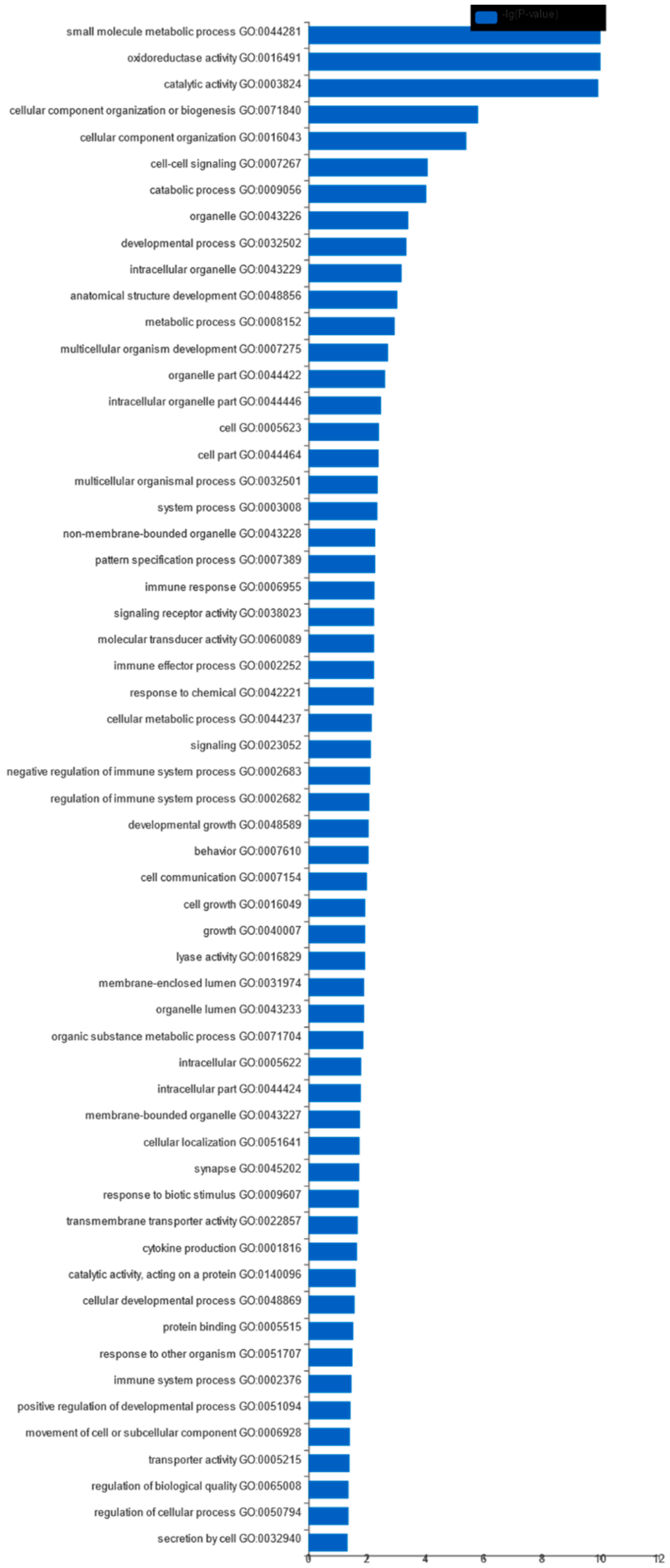


**Figure S7.** The Gene Ontology terms of the top 5% highly expressed transcripts that differ significantly ($\chi^2$ test p-value <0.05) between the liver and brain cerebral cortex cells.

**Table S2.** Definition and number of total KEGG metabolic pathways predicted in the brushtail possum liver transcriptome.

| Liver Metabolic Pathways | Number of Pathways |
|---|---|
| **Global and overview maps** | |
| 01100 Metabolic pathways | 506 |
| 01110 Biosynthesis of secondary metabolites | 143 |
| 01120 Microbial metabolism in diverse environments | 83 |
| 01130 Biosynthesis of antibiotics | 100 |
| 01200 Carbon metabolism | 55 |
| 01210 2-Oxocarboxylic acid metabolism | 7 |
| 01212 Fatty acid metabolism | 23 |
| 01230 Biosynthesis of amino acids | 27 |
| 01220 Degradation of aromatic compounds | 2 |
| **Carbohydrate metabolism** | |
| 00010 Glycolysis / Gluconeogenesis | 26 |
| 00020 Citrate cycle | 16 |
| 00030 Pentose phosphate pathway | 11 |
| 00040 Pentose and glucuronate interconversions | 11 |
| 00051 Fructose and mannose metabolism | 10 |
| 00052 Galactose metabolism | 11 |
| 00053 Ascorbate and aldarate metabolism | 6 |
| 00500 Starch and sucrose metabolism | 13 |
| 00520 Amino sugar and nucleotide sugar metabolism | 21 |
| 00620 Pyruvate metabolism | 24 |
| 00630 Glyoxylate and dicarboxylate metabolism | 20 |
| 00640 Propanoate metabolism | 22 |
| 00650 Butanoate metabolism | 11 |
| 00562 Inositol phosphate metabolism | 17 |
| **Energy metabolism** | |
| 00190 Oxidative phosphorylation | 50 |
| 00710 Carbon fixation in photosynthetic organisms | 10 |
| 00720 Carbon fixation pathways in prokaryotes | 6 |
| 00680 Methane metabolism | 11 |
| 00910 Nitrogen metabolism | 4 |
| 00920 Sulfur metabolism | 4 |
| **Lipid metabolism** | |
| 00061 Fatty acid biosynthesis | 7 |
| 00062 Fatty acid elongation | 7 |
| 00071 Fatty acid degradation | 20 |
| 00072 Synthesis and degradation of ketone bodies | 4 |
| 00100 Steroid biosynthesis | 11 |
| 00120 Primary bile acid biosynthesis | 12 |
| 00140 Steroid hormone biosynthesis | 20 |
| 00561 Glycerolipid metabolism | 20 |
| 00564 Glycerophospholipid metabolism | 25 |
| 00565 Ether lipid metabolism | 10 |
| 00600 Sphingolipid metabolism | 9 |
| 00590 Arachidonic acid metabolism | 13 |
| 00591 Linoleic acid metabolism | 8 |
| 00592 alpha-Linolenic acid metabolism | 2 |
| 01040 Biosynthesis of unsaturated fatty acids | 9 |

| Liver Metabolic Pathways | Number of Pathways |
| --- | --- |
| **Nucleotide metabolism** | |
| 00230 Purine metabolism | 36 |
| 00240 Pyrimidine metabolism | 16 |
| **Amino acid metabolism** | |
| 00250 Alanine, aspartate and glutamate metabolism | 9 |
| 00260 Glycine, serine and threonine metabolism | 21 |
| 00270 Cysteine and methionine metabolism | 23 |
| 00280 Valine, leucine and isoleucine degradation | 26 |
| 00300 Lysine biosynthesis | 1 |
| 00310 Lysine degradation | 26 |
| 00220 Arginine biosynthesis | 7 |
| 00330 Arginine and proline metabolism | 18 |
| 00340 Histidine metabolism | 7 |
| 00350 Tyrosine metabolism | 13 |
| 00360 Phenylalanine metabolism | 9 |
| 00380 Tryptophan metabolism | 22 |
| 00400 Phenylalanine, tyrosine and tryptophan biosynthesis | 3 |
| **Metabolism of other amino acids** | |
| 00410 beta-Alanine metabolism | 16 |
| 00430 Taurine and hypotaurine metabolism | 3 |
| 00440 Phosphonate and phosphinate metabolism | 3 |
| 00450 Selenocompound metabolism | 5 |
| 00460 Cyanoamino acid metabolism | 2 |
| 00471 D-Glutamine and D-glutamate metabolism | 1 |
| 00472 D-Arginine and D-ornithine metabolism | 1 |
| 00480 Glutathione metabolism | 13 |
| **Glycan biosynthesis and metabolism** | |
| 00510 N-Glycan biosynthesis | 20 |
| 00513 Various types of N-glycan biosynthesis | 14 |
| 00512 Mucin type O-glycan biosynthesis | 4 |
| 00515 Mannose type O-glycan biosynthesis | 2 |
| 00514 Other types of O-glycan biosynthesis | 3 |
| 00532 Glycosaminoglycan biosynthesis - chondroitin sulfate / dermatan sulfate | 3 |
| 00534 Glycosaminoglycan biosynthesis - heparan sulfate / heparin | 7 |
| 00533 Glycosaminoglycan biosynthesis - keratan sulfate | 3 |
| 00531 Glycosaminoglycan degradation | 7 |
| 00563 Glycosylphosphatidylinositol | 10 |
| 00601 Glycosphingolipid biosynthesis - lacto and neolacto series | 5 |
| 00603 Glycosphingolipid biosynthesis - globo and isoglobo series | 3 |
| 00604 Glycosphingolipid biosynthesis - ganglio series | 5 |
| 00511 Other glycan degradation | 5 |
| **Metabolism of cofactors and vitamins** | |
| 00730 Thiamine metabolism | 3 |
| 00740 Riboflavin metabolism | 4 |
| 00750 Vitamin B6 metabolism | 3 |
| 00760 Nicotinate and nicotinamide metabolism | 15 |
| 00770 Pantothenate and CoA biosynthesis | 6 |
| 00780 Biotin metabolism | 3 |
| 00785 Lipoic acid metabolism | 1 |

| Liver Metabolic Pathways | Number of Pathways |
|---|---|
| 00790 Folate biosynthesis | 14 |
| 00670 One carbon pool by folate | 9 |
| 00830 Retinol metabolism | 23 |
| 00860 Porphyrin and chlorophyll metabolism | 13 |
| 00130 Ubiquinone and other terpenoid-quinone biosynthesis | 7 |
| **Metabolism of terpenoids and polyketides** | |
| 00900 Terpenoid backbone biosynthesis | 9 |
| 00909 Sesquiterpenoid and triterpenoid biosynthesis | 2 |
| 00981 Insect hormone biosynthesis | 1 |
| 00903 Limonene and pinene degradation | 1 |
| 00281 Geraniol degradation | 1 |
| 01051 Biosynthesis of ansamycins | 1 |
| 00523 Polyketide sugar unit biosynthesis | 1 |
| 01055 Biosynthesis of vancomycin group antibiotics | 1 |
| **Biosynthesis of other secondary metabolites** | |
| 00940 Phenylpropanoid biosynthesis | 1 |
| 00944 Flavone and flavonol biosynthesis | 1 |
| 00901 Indole alkaloid biosynthesis | 1 |
| 00950 Isoquinoline alkaloid biosynthesis | 4 |
| 00960 Tropane, piperidine and pyridine alkaloid biosynthesis | 2 |
| 00232 Caffeine metabolism | 3 |
| 00965 Betalain biosynthesis | 2 |
| 00311 Penicillin and cephalosporin biosynthesis | 1 |
| 00521 Streptomycin biosynthesis | 4 |
| 00524 Neomycin, kanamycin and gentamicin biosynthesis | 1 |
| 00525 Acarbose and validamycin biosynthesis | 1 |
| 00401 Novobiocin biosynthesis | 1 |
| 00254 Aflatoxin biosynthesis | 2 |
| **Xenobiotics biodegradation and metabolism** | |
| 00362 Benzoate degradation | 1 |
| 00627 Aminobenzoate degradation | 3 |
| 00364 Fluorobenzoate degradation | 1 |
| 00625 Chloroalkane and chloroalkene degradation | 3 |
| 00361 Chlorocyclohexane and chlorobenzene degradation | 1 |
| 00623 Toluene degradation | 1 |
| 00633 Nitrotoluene degradation | 1 |
| 00643 Styrene degradation | 2 |
| 00930 Caprolactam degradation | 4 |
| 00626 Naphthalene degradation | 1 |
| 00980 Metabolism of xenobiotics by cytochrome P450 | 17 |
| 00982 Drug metabolism - cytochrome P450 | 14 |
| 00983 Drug metabolism - other enzymes | 20 |
| **Genetic Information Processing** | |
| **Transcription** | |
| 03020 RNA polymerase | 8 |
| 03022 Basal transcription factors | 14 |
| 03040 Spliceosome | 36 |
| **Translation** | |
| 03010 Ribosome | 70 |
| 00970 Aminoacyl-tRNA biosynthesis | 14 |

| Liver Metabolic Pathways | Number of Pathways |
|---|---|
| 03013 RNA transport | 54 |
| 03015 mRNA surveillance pathway | 25 |
| 03008 Ribosome biogenesis in eukaryotes | 34 |
| **Folding, sorting and degradation** | |
| 03060 Protein export | 11 |
| 04141 Protein processing in endoplasmic reticulum | 78 |
| 04130 SNARE interactions in vesicular transport | 10 |
| 04120 Ubiquitin mediated proteolysis | 46 |
| 04122 Sulfur relay system | 3 |
| 03050 Proteasome | 26 |
| 03018 RNA degradation | 24 |
| **Replication and repair** | |
| 03030 DNA replication | 9 |
| 03410 Base excision repair | 7 |
| 03420 Nucleotide excision repair | 13 |
| 03430 Mismatch repair | 5 |
| 03440 Homologous recombination | 8 |
| 03450 Non-homologous end-joining | 4 |
| 03460 Fanconi anemia pathway | 9 |
| **Environmental Information Processing** | |
| **Membrane transport** | |
| 02010 ABC transporters | 13 |
| 03070 Bacterial secretion system | 1 |
| **Signal transduction** | |
| 02020 Two-component system | 3 |
| 04014 Ras signaling pathway | 44 |
| 04015 Rap1 signaling pathway | 48 |
| 04010 MAPK signaling pathway | 59 |
| 04013 MAPK signaling pathway - fly | 20 |
| 04016 MAPK signaling pathway - plant | 3 |
| 04011 MAPK signaling pathway - yeast | 6 |
| 04012 ErbB signaling pathway | 21 |
| 04310 Wnt signaling pathway | 27 |
| 04330 Notch signaling pathway | 12 |
| 04340 Hedgehog signaling pathway | 3 |
| 04341 Hedgehog signaling pathway - fly | 3 |
| 04350 TGF-beta signaling pathway | 28 |
| 04390 Hippo signaling pathway | 37 |
| 04391 Hippo signaling pathway - fly | 15 |
| 04392 Hippo signaling pathway - multiple species | 6 |
| 04370 VEGF signaling pathway | 16 |
| 04371 Apelin signaling pathway | 26 |
| 04630 JAK-STAT signaling pathway | 33 |
| 04064 NF-kappa B signaling pathway | 22 |
| 04668 TNF signaling pathway | 23 |
| 04066 HIF-1 signaling pathway | 33 |
| 04068 FoxO signaling pathway | 33 |
| 04020 Calcium signaling pathway | 24 |
| 04070 Phosphatidylinositol signaling system | 18 |
| 04072 Phospholipase D signaling pathway | 29 |

| Liver Metabolic Pathways | Number of Pathways |
|---|---|
| 04071 Sphingolipid signaling pathway | 33 |
| 04024 cAMP signaling pathway | 31 |
| 04022 cGMP-PKG signaling pathway | 39 |
| 04151 PI3K-Akt signaling pathway | 80 |
| 04152 AMPK signaling pathway | 40 |
| 04150 mTOR signaling pathway | 34 |
| **Signaling molecules and interaction** | |
| 04080 Neuroactive ligand-receptor interaction | 20 |
| 04060 Cytokine-cytokine receptor interaction | 40 |
| 04061 Viral protein interaction with cytokine and cytokine receptor | 15 |
| 04512 ECM-receptor interaction | 19 |
| 04514 Cell adhesion molecules | 23 |
| **Cellular Processes** | 980 |
| **Transport and catabolism** | |
| 04144 Endocytosis | 73 |
| 04145 Phagosome | 46 |
| 04142 Lysosome | 51 |
| 04146 Peroxisome | 47 |
| 04140 Autophagy - animal | 41 |
| 04138 Autophagy - yeast | 16 |
| 04136 Autophagy - other | 8 |
| 04137 Mitophagy - animal | 18 |
| 04139 Mitophagy - yeast | 10 |
| **Cell growth and death** | |
| 04110 Cell cycle | 28 |
| 04111 Cell cycle - yeast | 22 |
| 04112 Cell cycle - Caulobacter | 2 |
| 04113 Meiosis - yeast | 16 |
| 04114 Oocyte meiosis | 24 |
| 04210 Apoptosis | 40 |
| 04214 Apoptosis - fly | 15 |
| 04215 Apoptosis - multiple species | 7 |
| 04216 Ferroptosis | 15 |
| 04217 Necroptosis | 39 |
| 04115 p53 signaling pathway | 16 |
| 04218 Cellular senescence | 45 |
| **Cellular community - eukaryotes** | |
| 04510 Focal adhesion | 55 |
| 04520 Adherens junction | 26 |
| 04530 Tight junction | 38 |
| 04540 Gap junction | 13 |
| **04550 Signaling pathways regulating pluripotency of stem cells** | 30 |
| Cellular community - prokaryotes | |
| 02024 Quorum sensing | 2 |
| 02026 Biofilm formation - Escherichia coli | 1 |
| **Cell motility** | |
| 04810 Regulation of actin cytoskeleton | 57 |
| **Organismal Systems** | |
| **Immune system** | |
| 04640 Hematopoietic cell lineage | 17 |

| Liver Metabolic Pathways | Number of Pathways |
|---|---|
| 04610 Complement and coagulation cascades | 43 |
| 04611 Platelet activation | 31 |
| 04620 Toll-like receptor signaling pathway | 29 |
| 04624 Toll and Imd signaling pathway | 11 |
| 04621 NOD-like receptor signaling pathway | 42 |
| 04622 RIG-I-like receptor signaling pathway | 15 |
| 04623 Cytosolic DNA-sensing pathway | 14 |
| 04625 C-type lectin receptor signaling pathway | 25 |
| 04650 Natural killer cell mediated cytotoxicity | 22 |
| 04612 Antigen processing and presentation | 12 |
| 04660 T cell receptor signaling pathway | 23 |
| 04658 Th1 and Th2 cell differentiation | 21 |
| 04659 Th17 cell differentiation | 27 |
| 04657 IL-17 signaling pathway | 16 |
| 04662 B cell receptor signaling pathway | 27 |
| 04664 Fc epsilon RI signaling pathway | 17 |
| 04666 Fc gamma R-mediated phagocytosis | 22 |
| 04670 Leukocyte transendothelial migration | 30 |
| 04672 Intestinal immune network for IgA production | 6 |
| 04062 Chemokine signaling pathway | 37 |
| **Endocrine system** | |
| 04911 Insulin secretion | 11 |
| 04910 Insulin signaling pathway | 42 |
| 04922 Glucagon signaling pathway | 34 |
| 04923 Regulation of lipolysis in adipocytes | 8 |
| 04920 Adipocytokine signaling pathway | 24 |
| 03320 PPAR signaling pathway | 25 |
| 04929 GnRH secretion | 11 |
| 04912 GnRH signaling pathway | 15 |
| 04913 Ovarian steroidogenesis | 12 |
| 04915 Estrogen signaling pathway | 24 |
| 04914 Progesterone-mediated oocyte maturation | 16 |
| 04917 Prolactin signaling pathway | 17 |
| 04921 Oxytocin signaling pathway | 26 |
| 04926 Relaxin signaling pathway | 25 |
| 04935 Growth hormone synthesis, secretion and action | 27 |
| 04918 Thyroid hormone synthesis | 15 |
| 04919 Thyroid hormone signaling pathway | 35 |
| 04928 Parathyroid hormone synthesis, secretion and action | 16 |
| 04916 Melanogenesis | 15 |
| 04924 Renin secretion | 11 |
| 04614 Renin-angiotensin system | 7 |
| 04925 Aldosterone synthesis and secretion | 15 |
| 04927 Cortisol synthesis and secretion | 12 |
| **Circulatory system** | |
| 04260 Cardiac muscle contraction | 12 |
| 04261 Adrenergic signaling in cardiomyocytes | 19 |
| 04270 Vascular smooth muscle contraction | 22 |
| **Digestive system** | |
| 04970 Salivary secretion | 14 |

| Liver Metabolic Pathways | Number of Pathways |
|---|---|
| 04971 Gastric acid secretion | 12 |
| 04972 Pancreatic secretion | 17 |
| 04976 Bile secretion | 22 |
| 04973 Carbohydrate digestion and absorption | 5 |
| 04974 Protein digestion and absorption | 13 |
| 04975 Fat digestion and absorption | 10 |
| 04979 Cholesterol metabolism | 20 |
| 04977 Vitamin digestion and absorption | 7 |
| 04978 Mineral absorption | 9 |
| **Excretory system** | |
| 04962 Vasopressin-regulated water reabsorption | 12 |
| 04960 Aldosterone-regulated sodium reabsorption | 7 |
| 04961 Endocrine and other factor-regulated calcium reabsorption | 10 |
| 04964 Proximal tubule bicarbonate reclamation | 8 |
| 04966 Collecting duct acid secretion | 9 |
| **Nervous system** | |
| 04724 Glutamatergic synapse | 11 |
| 04727 GABAergic synapse | 7 |
| 04725 Cholinergic synapse | 12 |
| 04728 Dopaminergic synapse | 19 |
| 04726 Serotonergic synapse | 15 |
| 04720 Long-term potentiation | 16 |
| 04730 Long-term depression | 11 |
| 04723 Retrograde endocannabinoid signaling | 27 |
| 04721 Synaptic vesicle cycle | 14 |
| 04722 Neurotrophin signaling pathway | 28 |
| **Sensory system** | |
| 04744 Phototransduction | 1 |
| 04745 Phototransduction - fly | 5 |
| 04740 Olfactory transduction | 1 |
| 04742 Taste transduction | 2 |
| 04750 Inflammatory mediator regulation of TRP channels | 14 |
| **Development and regeneration** | |
| 04320 Dorso-ventral axis formation | 6 |
| 04360 Axon guidance | 32 |
| 04361 Axon regeneration | 20 |
| 04380 Osteoclast differentiation | 31 |
| **Aging** | |
| 04211 Longevity regulating pathway | 26 |
| 04212 Longevity regulating pathway - worm | 24 |
| 04213 Longevity regulating pathway - multiple species | 18 |
| **Environmental adaptation** | |
| 04710 Circadian rhythm | 9 |
| 04713 Circadian entrainment | 6 |
| 04711 Circadian rhythm - fly | 2 |
| 04714 Thermogenesis | 71 |
| 04626 Plant-pathogen interaction | 6 |

**Table S3.** Definition and number of total KEGG metabolic pathways predicted in the brushtail possum brain cerebral cortex transcriptome.

| Brain Metabolic Pathways | Number of Pathways |
|---|---|
| **Global and overview maps** | |
| 01100 Metabolic pathways | 301 |
| 01110 Biosynthesis of secondary metabolites | 89 |
| 01120 Microbial metabolism in diverse environments | 45 |
| 01130 Biosynthesis of antibiotics | 60 |
| 01200 Carbon metabolism | 33 |
| 01210 2-Oxocarboxylic acid metabolism | 7 |
| 01212 Fatty acid metabolism | 22 |
| 01230 Biosynthesis of amino acids | 18 |
| 01220 Degradation of aromatic compounds | 2 |
| **Carbohydrate metabolism** | |
| 00010 Glycolysis / Gluconeogenesis | 15 |
| 00020 Citrate cycle | 14 |
| 00030 Pentose phosphate pathway | 5 |
| 00040 Pentose and glucuronate interconversions | 4 |
| 00051 Fructose and mannose metabolism | 3 |
| 00052 Galactose metabolism | 4 |
| 00053 Ascorbate and aldarate metabolism | 3 |
| 00500 Starch and sucrose metabolism | 8 |
| 00520 Amino sugar and nucleotide sugar metabolism | 9 |
| 00620 Pyruvate metabolism | 11 |
| 00630 Glyoxylate and dicarboxylate metabolism | 6 |
| 00640 Propanoate metabolism | 8 |
| 00650 Butanoate metabolism | 8 |
| 00562 Inositol phosphate metabolism | 14 |
| **Energy metabolism** | |
| 00190 Oxidative phosphorylation | 41 |
| 00710 Carbon fixation in photosynthetic organisms | 8 |
| 00720 Carbon fixation pathways in prokaryotes | 4 |
| 00680 Methane metabolism | 5 |
| 00910 Nitrogen metabolism | 2 |
| 00920 Sulfur metabolism | 3 |
| **Lipid metabolism** | |
| 00061 Fatty acid biosynthesis | 4 |
| 00062 Fatty acid elongation | 10 |
| 00071 Fatty acid degradation | 14 |
| 00072 Synthesis and degradation of ketone bodies | 1 |
| 00073 Cutin, suberine and wax biosynthesis | 1 |
| 00100 Steroid biosynthesis | 3 |
| 00120 Primary bile acid biosynthesis | 4 |
| 00140 Steroid hormone biosynthesis | 3 |
| 00561 Glycerolipid metabolism | 11 |
| 00564 Glycerophospholipid metabolism | 19 |
| 00565 Ether lipid metabolism | 10 |
| 00600 Sphingolipid metabolism | 12 |
| 00590 Arachidonic acid metabolism | 5 |
| 00591 Linoleic acid metabolism | 1 |
| 00592 alpha-Linolenic acid metabolism | 1 |
| 01040 Biosynthesis of unsaturated fatty acids | 9 |

| Brain Metabolic Pathways | Number of Pathways |
|---|---|
| **Nucleotide metabolism** | |
| 00230 Purine metabolism | 28 |
| 00240 Pyrimidine metabolism | 5 |
| **Amino acid metabolism** | |
| 00250 Alanine, aspartate and glutamate metabolism | 10 |
| 00260 Glycine, serine and threonine metabolism | 5 |
| 00270 Cysteine and methionine metabolism | 12 |
| 00280 Valine, leucine and isoleucine degradation | 16 |
| 00300 Lysine biosynthesis | 1 |
| 00310 Lysine degradation | 16 |
| 00220 Arginine biosynthesis | 7 |
| 00330 Arginine and proline metabolism | 15 |
| 00340 Histidine metabolism | 2 |
| 00350 Tyrosine metabolism | 5 |
| 00360 Phenylalanine metabolism | 4 |
| 00380 Tryptophan metabolism | 8 |
| 00400 Phenylalanine, tyrosine and tryptophan biosynthesis | 2 |
| **Metabolism of other amino acids** | |
| 00410 beta-Alanine metabolism | 7 |
| 00430 Taurine and hypotaurine metabolism | 1 |
| 00440 Phosphonate and phosphinate metabolism | 2 |
| 00450 Selenocompound metabolism | 4 |
| 00460 Cyanoamino acid metabolism | 1 |
| 00471 D-Glutamine and D-glutamate metabolism | 1 |
| 00480 Glutathione metabolism | 7 |
| **Glycan biosynthesis and metabolism** | |
| 00510 N-Glycan biosynthesis | 14 |
| 00513 Various types of N-glycan biosynthesis | 11 |
| 00512 Mucin type O-glycan biosynthesis | 2 |
| 00515 Mannose type O-glycan biosynthesis | 7 |
| 00514 Other types of O-glycan biosynthesis | 3 |
| 00532 Glycosaminoglycan biosynthesis - chondroitin sulfate / dermatan sulfate | 1 |
| 00534 Glycosaminoglycan biosynthesis - heparan sulfate / heparin | 10 |
| 00533 Glycosaminoglycan biosynthesis - keratan sulfate | 3 |
| 00531 Glycosaminoglycan degradation | 4 |
| 00563 Glycosylphosphatidylinositol | 9 |
| 00601 Glycosphingolipid biosynthesis - lacto and neolacto series | 4 |
| 00603 Glycosphingolipid biosynthesis - globo and isoglobo series | 2 |
| 00604 Glycosphingolipid biosynthesis - ganglio series | 7 |
| 00511 Other glycan degradation | 7 |
| **Metabolism of cofactors and vitamins** | |
| 00730 Thiamine metabolism | 3 |
| 00740 Riboflavin metabolism | 1 |
| 00750 Vitamin B6 metabolism | 2 |
| 00760 Nicotinate and nicotinamide metabolism | 5 |
| 00770 Pantothenate and CoA biosynthesis | 1 |
| 00785 Lipoic acid metabolism | 1 |
| 00790 Folate biosynthesis | 4 |
| 00670 One carbon pool by folate | 3 |
| 00830 Retinol metabolism | 5 |
| 00860 Porphyrin and chlorophyll metabolism | 4 |

| Brain Metabolic Pathways | Number of Pathways |
|---|---|
| 00130 Ubiquinone and other terpenoid-quinone biosynthesis | 3 |
| **Metabolism of terpenoids and polyketides** | |
| 00900 Terpenoid backbone biosynthesis | 4 |
| 00981 Insect hormone biosynthesis | 1 |
| 00903 Limonene and pinene degradation | 1 |
| 01051 Biosynthesis of ansamycins | 1 |
| **Biosynthesis of other secondary metabolites** | |
| 00940 Phenylpropanoid biosynthesis | 1 |
| 00950 Isoquinoline alkaloid biosynthesis | 3 |
| 00960 Tropane, piperidine and pyridine alkaloid biosynthesis | 2 |
| 00261 Monobactam biosynthesis | 1 |
| 00521 Streptomycin biosynthesis | 1 |
| 00524 Neomycin, kanamycin and gentamicin biosynthesis | 1 |
| **Xenobiotics biodegradation and metabolism** | |
| 00362 Benzoate degradation | 1 |
| 00627 Aminobenzoate degradation | 4 |
| 00364 Fluorobenzoate degradation | 1 |
| 00625 Chloroalkane and chloroalkene degradation | 2 |
| 00361 Chlorocyclohexane and chlorobenzene degradation | 1 |
| 00623 Toluene degradation | 1 |
| 00930 Caprolactam degradation | 4 |
| 00626 Naphthalene degradation | 1 |
| 00980 Metabolism of xenobiotics by cytochrome P450 | 5 |
| 00982 Drug metabolism - cytochrome P450 | 4 |
| 00983 Drug metabolism - other enzymes | 6 |
| | |
| **Genetic Information Processing** | |
| **Transcription** | |
| 03020 RNA polymerase | 3 |
| 03022 Basal transcription factors | 10 |
| 03040 Spliceosome | 30 |
| **Translation** | |
| 03010 Ribosome | 55 |
| 00970 Aminoacyl-tRNA biosynthesis | 13 |
| 03013 RNA transport | 48 |
| 03015 mRNA surveillance pathway | 21 |
| 03008 Ribosome biogenesis in eukaryotes | 17 |
| **Folding, sorting and degradation** | |
| 03060 Protein export | 10 |
| 04141 Protein processing in endoplasmic reticulum | 53 |
| 04130 SNARE interactions in vesicular transport | 8 |
| 04120 Ubiquitin mediated proteolysis | 35 |
| 03050 Proteasome | 14 |
| 03018 RNA degradation | 15 |
| **Replication and repair** | |
| 03030 DNA replication | 4 |
| 03410 Base excision repair | 4 |
| 03420 Nucleotide excision repair | 10 |
| 03430 Mismatch repair | 4 |
| 03440 Homologous recombination | 2 |
| 03450 Non-homologous end-joining | 1 |

| Brain Metabolic Pathways | Number of Pathways |
|---|---|
| 03460 Fanconi anemia pathway | 7 |
| **Environmental Information Processing** | |
| **Membrane transport** | |
| 02010 ABC transporters | 9 |
| 03070 Bacterial secretion system | 1 |
| **Signal transduction** | |
| 02020 Two-component system | 4 |
| 04014 Ras signaling pathway | 52 |
| 04015 Rap1 signaling pathway | 55 |
| 04010 MAPK signaling pathway | 56 |
| 04013 MAPK signaling pathway - fly | 21 |
| 04016 MAPK signaling pathway - plant | 3 |
| 04011 MAPK signaling pathway - yeast | 7 |
| 04012 ErbB signaling pathway | 22 |
| 04310 Wnt signaling pathway | 19 |
| 04330 Notch signaling pathway | 8 |
| 04340 Hedgehog signaling pathway | 4 |
| 04341 Hedgehog signaling pathway - fly | 2 |
| 04350 TGF-beta signaling pathway | 13 |
| 04390 Hippo signaling pathway | 23 |
| 04391 Hippo signaling pathway - fly | 13 |
| 04392 Hippo signaling pathway - multiple species | 2 |
| 04370 VEGF signaling pathway | 12 |
| 04371 Apelin signaling pathway | 36 |
| 04630 JAK-STAT signaling pathway | 18 |
| 04064 NF-kappa B signaling pathway | 5 |
| 04668 TNF signaling pathway | 15 |
| 04066 HIF-1 signaling pathway | 27 |
| 04068 FoxO signaling pathway | 29 |
| 04020 Calcium signaling pathway | 32 |
| 04070 Phosphatidylinositol signaling system | 20 |
| 04072 Phospholipase D signaling pathway | 37 |
| 04071 Sphingolipid signaling pathway | 30 |
| 04024 cAMP signaling pathway | 45 |
| 04022 cGMP-PKG signaling pathway | 36 |
| 04151 PI3K-Akt signaling pathway | 63 |
| 04152 AMPK signaling pathway | 27 |
| 04150 mTOR signaling pathway | 29 |
| **Signaling molecules and interaction** | |
| 04080 Neuroactive ligand-receptor interaction | 24 |
| 04060 Cytokine-cytokine receptor interaction | 15 |
| 04061 Viral protein interaction with cytokine and cytokine receptor | 8 |
| 04512 ECM-receptor interaction | 10 |
| 04514 Cell adhesion molecules | 22 |
| **Cellular Processes** | |
| **Transport and catabolism** | |
| 04144 Endocytosis | 71 |
| 04145 Phagosome | 32 |
| 04142 Lysosome | 35 |
| 04146 Peroxisome | 21 |
| 04140 Autophagy - animal | 41 |

| Brain Metabolic Pathways | Number of Pathways |
|---|---|
| 04138 Autophagy - yeast | 20 |
| 04136 Autophagy - other | 13 |
| 04137 Mitophagy - animal | 19 |
| 04139 Mitophagy - yeast | 10 |
| **Cell growth and death** | |
| 04110 Cell cycle | 16 |
| 04111 Cell cycle - yeast | 10 |
| 04113 Meiosis - yeast | 9 |
| 04114 Oocyte meiosis | 22 |
| 04210 Apoptosis | 25 |
| 04214 Apoptosis - fly | 13 |
| 04215 Apoptosis - multiple species | 4 |
| 04216 Ferroptosis | 9 |
| 04217 Necroptosis | 21 |
| 04115 p53 signaling pathway | 11 |
| 04218 Cellular senescence | 33 |
| **Cellular community - eukaryotes** | |
| 04510 Focal adhesion | 42 |
| 04520 Adherens junction | 24 |
| 04530 Tight junction | 36 |
| 04540 Gap junction | 20 |
| 04550 Signaling pathways regulating pluripotency of stem cells | 22 |
| **Cellular community - prokaryotes** | |
| 02024 Quorum sensing | 3 |
| 02026 Biofilm formation - Escherichia coli | 1 |
| **Cell motility** | |
| 04810 Regulation of actin cytoskeleton | 46 |
| **Organismal Systems** | |
| **Immune system** | |
| 04640 Hematopoietic cell lineage | 10 |
| 04610 Complement and coagulation cascades | 10 |
| 04611 Platelet activation | 26 |
| 04620 Toll-like receptor signaling pathway | 12 |
| 04624 Toll and Imd signaling pathway | 4 |
| 04621 NOD-like receptor signaling pathway | 24 |
| 04622 RIG-I-like receptor signaling pathway | 6 |
| 04623 Cytosolic DNA-sensing pathway | 5 |
| 04625 C-type lectin receptor signaling pathway | 16 |
| 04650 Natural killer cell mediated cytotoxicity | 16 |
| 04612 Antigen processing and presentation | 8 |
| 04660 T cell receptor signaling pathway | 16 |
| 04658 Th1 and Th2 cell differentiation | 8 |
| 04659 Th17 cell differentiation | 13 |
| 04657 IL-17 signaling pathway | 8 |
| 04662 B cell receptor signaling pathway | 13 |
| 04664 Fc epsilon RI signaling pathway | 15 |
| 04666 Fc gamma R-mediated phagocytosis | 20 |
| 04670 Leukocyte transendothelial migration | 21 |
| 04672 Intestinal immune network for IgA production | 1 |
| 04062 Chemokine signaling pathway | 34 |
| **Endocrine system** | |

| Brain Metabolic Pathways | Number of Pathways |
|---|---|
| 04911 Insulin secretion | 19 |
| 04910 Insulin signaling pathway | 30 |
| 04922 Glucagon signaling pathway | 23 |
| 04923 Regulation of lipolysis in adipocytes | 13 |
| 04920 Adipocytokine signaling pathway | 12 |
| 03320 PPAR signaling pathway | 13 |
| 04929 GnRH secretion | 15 |
| 04912 GnRH signaling pathway | 23 |
| 04913 Ovarian steroidogenesis | 6 |
| 04915 Estrogen signaling pathway | 30 |
| 04914 Progesterone-mediated oocyte maturation | 18 |
| 04917 Prolactin signaling pathway | 11 |
| 04921 Oxytocin signaling pathway | 32 |
| 04926 Relaxin signaling pathway | 28 |
| 04935 Growth hormone synthesis, secretion and action | 30 |
| 04918 Thyroid hormone synthesis | 18 |
| 04919 Thyroid hormone signaling pathway | 32 |
| 04928 Parathyroid hormone synthesis, secretion and action | 18 |
| 04916 Melanogenesis | 19 |
| 04924 Renin secretion | 14 |
| 04614 Renin-angiotensin system | 5 |
| 04925 Aldosterone synthesis and secretion | 21 |
| 04927 Cortisol synthesis and secretion | 15 |
| **Circulatory system** | |
| 04260 Cardiac muscle contraction | 21 |
| 04261 Adrenergic signaling in cardiomyocytes | 30 |
| 04270 Vascular smooth muscle contraction | 24 |
| **Digestive system** | |
| 04970 Salivary secretion | 18 |
| 04971 Gastric acid secretion | 20 |
| 04972 Pancreatic secretion | 21 |
| 04976 Bile secretion | 18 |
| 04973 Carbohydrate digestion and absorption | 9 |
| 04974 Protein digestion and absorption | 4 |
| 04975 Fat digestion and absorption | 5 |
| 04979 Cholesterol metabolism | 12 |
| 04977 Vitamin digestion and absorption | 2 |
| 04978 Mineral absorption | 8 |
| **Excretory system** | |
| 04962 Vasopressin-regulated water reabsorption | 11 |
| 04960 Aldosterone-regulated sodium reabsorption | 11 |
| 04961 Endocrine and other factor-regulated calcium reabsorption | 15 |
| 04964 Proximal tubule bicarbonate reclamation | 5 |
| 04966 Collecting duct acid secretion | 7 |
| **Nervous system** | |
| 04724 Glutamatergic synapse | 31 |
| 04727 GABAergic synapse | 24 |
| 04725 Cholinergic synapse | 25 |
| 04728 Dopaminergic synapse | 32 |
| 04726 Serotonergic synapse | 18 |
| 04720 Long-term potentiation | 21 |

| Brain Metabolic Pathways | Number of Pathways |
|---|---|
| 04730 Long-term depression | 16 |
| 04723 Retrograde endocannabinoid signaling | 41 |
| 04721 Synaptic vesicle cycle | 19 |
| 04722 Neurotrophin signaling pathway | 28 |
| **Sensory system** | |
| 04744 Phototransduction | 1 |
| 04745 Phototransduction - fly | 5 |
| 04740 Olfactory transduction | 6 |
| 04742 Taste transduction | 8 |
| 04750 Inflammatory mediator regulation of TRP channels | 19 |
| **Development and regeneration** | |
| 04320 Dorso-ventral axis formation | 7 |
| 04360 Axon guidance | 34 |
| 04361 Axon regeneration | 23 |
| 04380 Osteoclast differentiation | 23 |
| **Aging** | |
| 04211 Longevity regulating pathway | 26 |
| 04212 Longevity regulating pathway - worm | 22 |
| 04213 Longevity regulating pathway - multiple species | 17 |
| Environmental adaptation | |
| 04710 Circadian rhythm | 5 |
| 04713 Circadian entrainment | 27 |
| 04711 Circadian rhythm - fly | 2 |
| 04712 Circadian rhythm - plant | 1 |
| 04714 Thermogenesis | 62 |
| 04626 Plant-pathogen interaction | 6 |

**Table S4.** Liver synonymous/non-synonymous amino acid substitution.

| Number of occurrences | Codon change | Amino acid change | | Non-synonymous |
|---|---|---|---|---|
| 648 | GAC_GAT | D | D | FALSE |
| 630 | AAC_AAT | N | N | FALSE |
| 582 | GAT_GAC | D | D | FALSE |
| 552 | GAA_GAG | E | E | FALSE |
| 549 | ACA_ACG | T | T | FALSE |
| 519 | AAT_AAC | N | N | FALSE |
| 483 | ATT_ATC | I | I | FALSE |
| 480 | TAC_TAT | Y | Y | FALSE |
| 465 | CAC_CAT | H | H | FALSE |
| 459 | CCA_CCG | P | P | FALSE |
| 456 | GCA_GCG | A | A | FALSE |
| 426 | TCC_TCT | S | S | FALSE |
| 426 | GAG_GAA | E | E | FALSE |
| 426 | AAA_AAG | K | K | FALSE |
| 405 | ACC_ACT | T | T | FALSE |
| 393 | ACT_ACC | T | T | FALSE |
| 390 | GCC_GCT | A | A | FALSE |
| 381 | CTG_TTG | L | L | FALSE |
| 378 | GCG_GCA | A | A | FALSE |
| 378 | CCG_CCA | P | P | FALSE |
| 378 | AAG_AAA | K | K | FALSE |
| 375 | ACG_ACA | T | T | FALSE |

| Number of occurrences | Codon change | Amino acid change | | Non-synonymous |
|---|---|---|---|---|
| 372 | CAT_CAC | H | H | FALSE |
| 372 | CAA_CAG | Q | Q | FALSE |
| 363 | TAT_TAC | Y | Y | FALSE |
| 360 | TTG_CTG | L | L | FALSE |
| 360 | CTG_CTA | L | L | FALSE |
| 357 | ATC_ATT | I | I | FALSE |
| 342 | CAG_CAA | Q | Q | FALSE |
| 339 | GTG_GTA | V | V | FALSE |
| 318 | GGT_GGC | G | G | FALSE |
| 315 | TCA_TCG | S | S | FALSE |
| 300 | GCT_GCC | A | A | FALSE |
| 288 | GGC_GGT | G | G | FALSE |
| 285 | TCG_TCA | S | S | FALSE |
| 285 | GTT_GTC | V | V | FALSE |
| 276 | AGC_AGT | S | S | FALSE |
| 273 | CTA_CTG | L | L | FALSE |
| 261 | TTT_TTC | F | F | FALSE |
| 261 | CCC_CCT | P | P | FALSE |
| 252 | CTC_CTT | L | L | FALSE |
| 249 | GGG_GGA | G | G | FALSE |
| 231 | TTC_TTT | F | F | FALSE |
| 231 | CCT_CCC | P | P | FALSE |
| 225 | TGC_TGT | C | C | FALSE |
| 198 | TTG_TTA | L | L | FALSE |
| 198 | CTT_CTC | L | L | FALSE |
| 195 | GTC_GTT | V | V | FALSE |
| 192 | GGA_GGG | G | G | FALSE |
| 186 | TCT_TCC | S | S | FALSE |
| 183 | AGT_AGC | S | S | FALSE |
| 180 | TGT_TGC | C | C | FALSE |
| 171 | GTA_GTG | V | V | FALSE |
| 168 | AGA_AGG | R | R | FALSE |
| 153 | GTG_GTT | V | V | FALSE |
| 144 | AGG_AGA | R | R | FALSE |
| 141 | GCC_GCA | A | A | FALSE |
| 120 | GGC_GGA | G | G | FALSE |
| 114 | ACA_ACT | T | T | FALSE |
| 105 | TTA_TTG | L | L | FALSE |
| 99 | CTC_CTG | L | L | FALSE |
| 96 | CTG_CTC | L | L | FALSE |
| 96 | ATC_ATA | I | I | FALSE |
| 96 | ACG_ACT | T | T | FALSE |
| 93 | CGT_CGC | R | R | FALSE |
| 90 | CGC_CGT | R | R | FALSE |
| 90 | CCC_CCA | P | P | FALSE |
| 87 | GGC_GGG | G | G | FALSE |
| 84 | ACC_ACA | T | T | FALSE |
| 81 | GGA_GGC | G | G | FALSE |
| 81 | GCG_GCT | A | A | FALSE |
| 81 | CTG_CTT | L | L | FALSE |
| 81 | CTA_TTA | L | L | FALSE |

| Number of occurrences | Codon change | Amino acid change | | Non-synonymous |
|---|---|---|---|---|
| 81 | ACG_ACC | T | T | FALSE |
| 78 | TCC_TCA | S | S | FALSE |
| 78 | GTC_GTG | V | V | FALSE |
| 75 | CCC_CCG | P | P | FALSE |
| 72 | TTA_CTA | L | L | FALSE |
| 72 | CCG_CCT | P | P | FALSE |
| 69 | GTG_GTC | V | V | FALSE |
| 66 | GGG_GGT | G | G | FALSE |
| 63 | CTC_CTA | L | L | FALSE |
| 60 | TCC_TCG | S | S | FALSE |
| 57 | TCA_TCT | S | S | FALSE |
| 57 | GGG_GGC | G | G | FALSE |
| 51 | GTT_GTG | V | V | FALSE |
| 48 | GTT_GTA | V | V | FALSE |
| 48 | ACC_ACG | T | T | FALSE |
| 45 | GGA_GGT | G | G | FALSE |
| 45 | GCC_GCG | A | A | FALSE |
| 45 | CGG_CGA | R | R | FALSE |
| 42 | GTC_GTA | V | V | FALSE |
| 42 | CTT_CTG | L | L | FALSE |
| 42 | CCA_CCT | P | P | FALSE |
| 39 | GCA_GCT | A | A | FALSE |
| 39 | CGA_CGG | R | R | FALSE |
| 39 | ACT_ACA | T | T | FALSE |
| 39 | ACA_ACC | T | T | FALSE |
| 36 | GCT_GCA | A | A | FALSE |
| 36 | CGA_CGT | R | R | FALSE |
| 33 | TCG_TCC | S | S | FALSE |
| 33 | CGA_AGA | R | R | FALSE |
| 30 | CGG_AGG | R | R | FALSE |
| 30 | CGC_CGG | R | R | FALSE |
| 30 | ATT_ATA | I | I | FALSE |
| 27 | GCT_GCG | A | A | FALSE |
| 27 | CTT_CTA | L | L | FALSE |
| 27 | ATA_ATT | I | I | FALSE |
| 24 | GGT_GGA | G | G | FALSE |
| 24 | CGT_CGA | R | R | FALSE |
| 24 | CGG_CGC | R | R | FALSE |
| 24 | CCG_CCC | P | P | FALSE |
| 24 | AGA_CGA | R | R | FALSE |
| 21 | GCG_GCC | A | A | FALSE |
| 21 | CCA_CCC | P | P | FALSE |
| 18 | TCT_TCA | S | S | FALSE |
| 18 | TCG_TCT | S | S | FALSE |
| 15 | TCA_TCC | S | S | FALSE |
| 15 | GTA_GTT | V | V | FALSE |
| 15 | GGT_GGG | G | G | FALSE |
| 15 | CGG_CGT | R | R | FALSE |
| 15 | CGA_CGC | R | R | FALSE |
| 15 | CCT_CCG | P | P | FALSE |
| 12 | GCA_GCC | A | A | FALSE |

| Number of occurrences | Codon change | Amino acid change | | Non-synonymous |
|---|---|---|---|---|
| 12 | CTA_CTT | L | L | FALSE |
| 12 | CTA_CTC | L | L | FALSE |
| 12 | CGC_CGA | R | R | FALSE |
| 12 | ACT_ACG | T | T | FALSE |
| 9 | TCT_TCG | S | S | FALSE |
| 9 | N/A_N/A | L | L | FALSE |
| 9 | GTA_GTC | V | V | FALSE |
| 9 | CGT_CGG | R | R | FALSE |
| 9 | CCT_CCA | P | P | FALSE |
| 9 | ATC_GTC | A | A | FALSE |
| 9 | ATA_ATC | I | I | FALSE |
| 6 | TTC_CTC | E | E | FALSE |
| 6 | N/A_N/A | V | V | FALSE |
| 6 | N/A_N/A | Q | Q | FALSE |
| 6 | GGT_AGT | T | T | FALSE |
| 6 | GCT_ACT | S | S | FALSE |
| 6 | AGG_CGG | R | R | FALSE |
| 3 | N/A_N/A | T | T | FALSE |
| 3 | N/A_N/A | R | R | FALSE |
| 3 | N/A_N/A | N | N | FALSE |
| 3 | N/A_N/A | I | I | FALSE |
| 3 | N/A_N/A | E | E | FALSE |
| 3 | N/A_N/A | A | A | FALSE |
| 3 | CCA_CCG | T | T | FALSE |
| 3 | AAG_AAA | G | G | FALSE |
| 285 | GTT_ATT | V | I | TRUE |
| 234 | ACG_ATG | T | M | TRUE |
| 225 | GTC_ATC | V | I | TRUE |
| 192 | ATG_ACG | M | T | TRUE |
| 174 | AGT_AAT | S | N | TRUE |
| 159 | AAT_AGT | N | S | TRUE |
| 153 | GTA_ATA | V | I | TRUE |
| 153 | CAA_CGA | Q | R | TRUE |
| 141 | ATC_GTC | I | V | TRUE |
| 135 | ATT_GTT | I | V | TRUE |
| 132 | GTG_ATG | V | M | TRUE |
| 132 | ATG_GTG | M | V | TRUE |
| 126 | ATA_GTA | I | V | TRUE |
| 117 | GAT_AAT | D | N | TRUE |
| 117 | ACT_GCT | T | A | TRUE |
| 117 | ACA_GCA | T | A | TRUE |
| 114 | GCC_ACC | A | T | TRUE |
| 108 | GCA_ACA | A | T | TRUE |
| 108 | CAT_CGT | H | R | TRUE |
| 108 | ATG_ATA | M | I | TRUE |
| 108 | AAT_GAT | N | D | TRUE |
| 102 | AGA_AAA | R | K | TRUE |
| 99 | GTG_GCG | V | A | TRUE |
| 96 | CGT_CAT | R | H | TRUE |
| 96 | CAG_CGG | Q | R | TRUE |
| 96 | ATA_ACA | I | T | TRUE |

| Number of occurrences | Codon change | Amino acid change | | Non-synonymous |
|---|---|---|---|---|
| 93 | CGG_CAG | R | Q | TRUE |
| 90 | GAG_GAC | E | D | TRUE |
| 90 | ACC_AGC | T | S | TRUE |
| 90 | AAA_GAA | K | E | TRUE |
| 87 | CTT_TTT | L | F | TRUE |
| 87 | CAC_CGC | H | R | TRUE |
| 87 | AAG_AGG | K | R | TRUE |
| 87 | AAC_ACC | N | T | TRUE |
| 84 | CCT_GCT | P | A | TRUE |
| 81 | GTC_GCC | V | A | TRUE |
| 81 | CAT_TAT | H | Y | TRUE |
| 81 | AAA_AGA | K | R | TRUE |
| 78 | GAA_GGA | E | G | TRUE |
| 75 | GCT_ACT | A | T | TRUE |
| 75 | GCA_GTA | A | V | TRUE |
| 75 | GAG_AAG | E | K | TRUE |
| 75 | ATT_ACT | I | T | TRUE |
| 75 | ACC_GCC | T | A | TRUE |
| 75 | ACA_ATA | T | I | TRUE |
| 72 | GGT_AGT | G | S | TRUE |
| 72 | GAG_GAT | E | D | TRUE |
| 72 | ACC_AAC | T | N | TRUE |
| 66 | CTG_ATG | L | M | TRUE |
| 66 | ACT_ATT | T | I | TRUE |
| 66 | AAG_AAT | K | N | TRUE |
| 63 | TCT_TTT | S | F | TRUE |
| 63 | GGG_AGG | G | R | TRUE |
| 63 | GAC_GAA | D | E | TRUE |
| 60 | GCG_GTG | A | V | TRUE |
| 60 | GAA_AAA | E | K | TRUE |
| 57 | TGG_CGG | W | R | TRUE |
| 57 | TCG_TTG | S | L | TRUE |
| 54 | GCT_GTT | A | V | TRUE |
| 54 | CTC_TTC | L | F | TRUE |
| 54 | CCG_CTG | P | L | TRUE |
| 54 | AGG_AAG | R | K | TRUE |
| 54 | AAC_GAC | N | D | TRUE |
| 51 | TCA_TTA | S | L | TRUE |
| 51 | GCC_CCC | A | P | TRUE |
| 51 | GAC_AAC | D | N | TRUE |
| 51 | CTG_CAG | L | Q | TRUE |
| 51 | CGA_CAA | R | Q | TRUE |
| 51 | CCT_CTT | P | L | TRUE |
| 51 | AGT_ACT | S | T | TRUE |
| 48 | TTT_CTT | F | L | TRUE |
| 48 | GGA_AGA | G | R | TRUE |
| 48 | GCC_TCC | A | S | TRUE |
| 48 | CTG_CCG | L | P | TRUE |
| 48 | ATA_ATG | I | M | TRUE |
| 48 | ACC_ATC | T | I | TRUE |
| 48 | ACA_AGA | T | R | TRUE |

| Number of occurrences | Codon change | Amino acid change | | Non-synonymous |
|---|---|---|---|---|
| 45 | TTG_TCG | L | S | TRUE |
| 45 | TCA_ACA | S | T | TRUE |
| 45 | GGA_GAA | G | E | TRUE |
| 45 | GCT_TCT | A | S | TRUE |
| 45 | GCA_GGA | A | G | TRUE |
| 45 | CTC_ATC | L | I | TRUE |
| 45 | AGA_ACA | R | T | TRUE |
| 45 | AAG_GAG | K | E | TRUE |
| 42 | GGC_AGC | G | S | TRUE |
| 42 | CGT_TGT | R | C | TRUE |
| 42 | CAC_TAC | H | Y | TRUE |
| 42 | ATT_ATG | I | M | TRUE |
| 42 | AGT_GGT | S | G | TRUE |
| 42 | AGG_GGG | R | G | TRUE |
| 42 | AAA_CAA | K | Q | TRUE |
| 39 | TTC_CTC | F | L | TRUE |
| 39 | TTA_TCA | L | S | TRUE |
| 39 | TCT_CCT | S | P | TRUE |
| 39 | GCC_GTC | A | V | TRUE |
| 39 | GAG_CAG | E | Q | TRUE |
| 39 | CCT_TCT | P | S | TRUE |
| 39 | CCC_TCC | P | S | TRUE |
| 39 | ATC_ACC | I | T | TRUE |
| 39 | AGC_AAC | S | N | TRUE |
| 36 | GCC_GGC | A | G | TRUE |
| 36 | CCA_TCA | P | S | TRUE |
| 36 | CAG_GAG | Q | E | TRUE |
| 36 | CAA_GAA | Q | E | TRUE |
| 36 | AGC_ACC | S | T | TRUE |
| 36 | ACG_AGG | T | R | TRUE |
| 33 | TGT_CGT | C | R | TRUE |
| 33 | TGC_CGC | C | R | TRUE |
| 33 | TAT_TGT | Y | C | TRUE |
| 33 | TAT_CAT | Y | H | TRUE |
| 33 | TAC_TTC | Y | F | TRUE |
| 33 | TAC_CAC | Y | H | TRUE |
| 33 | GTT_TTT | V | F | TRUE |
| 33 | GTT_GCT | V | A | TRUE |
| 33 | GTC_CTC | V | L | TRUE |
| 33 | GCA_TCA | A | S | TRUE |
| 33 | CAA_AAA | Q | K | TRUE |
| 30 | TTG_GTG | L | V | TRUE |
| 30 | TTG_ATG | L | M | TRUE |
| 30 | TGT_TCT | C | S | TRUE |
| 30 | TCC_ACC | S | T | TRUE |
| 30 | TCA_CCA | S | P | TRUE |
| 30 | GAG_GTG | E | V | TRUE |
| 30 | CGT_CTT | R | L | TRUE |
| 30 | CGG_TGG | R | W | TRUE |
| 30 | CCA_CTA | P | L | TRUE |
| 30 | CAT_CTT | H | L | TRUE |

| Number of occurrences | Codon change | Amino acid change | | Non-synonymous |
|---|---|---|---|---|
| 30 | CAG_CAT | Q | H | TRUE |
| 30 | ATG_ATT | M | I | TRUE |
| 30 | ATG_AGG | M | R | TRUE |
| 30 | AAC_AGC | N | S | TRUE |
| 30 | AAC_AAG | N | K | TRUE |
| 27 | GCG_ACG | A | T | TRUE |
| 27 | GAT_GGT | D | G | TRUE |
| 27 | GAC_GAG | D | E | TRUE |
| 27 | CTT_CGT | L | R | TRUE |
| 27 | AAG_AAC | K | N | TRUE |
| 24 | TTT_TAT | F | Y | TRUE |
| 24 | TTT_GTT | F | V | TRUE |
| 24 | TTA_ATA | L | I | TRUE |
| 24 | TGT_TAT | C | Y | TRUE |
| 24 | GAG_GCG | E | A | TRUE |
| 24 | GAA_CAA | E | Q | TRUE |
| 24 | CTT_ATT | L | I | TRUE |
| 24 | CCC_CAC | P | H | TRUE |
| 24 | CAC_CAG | H | Q | TRUE |
| 24 | ATG_TTG | M | L | TRUE |
| 24 | AGC_GGC | S | G | TRUE |
| 24 | AGC_AGA | S | R | TRUE |
| 24 | ACT_AGT | T | S | TRUE |
| 21 | TTC_TCC | F | S | TRUE |
| 21 | TCT_GCT | S | A | TRUE |
| 21 | TCT_ACT | S | T | TRUE |
| 21 | TCC_TTC | S | F | TRUE |
| 21 | TCC_CCC | S | P | TRUE |
| 21 | GTG_CTG | V | L | TRUE |
| 21 | GGT_GAT | G | D | TRUE |
| 21 | GCA_CCA | A | P | TRUE |
| 21 | GAT_GAG | D | E | TRUE |
| 21 | GAC_TAC | D | Y | TRUE |
| 21 | GAC_GGC | D | G | TRUE |
| 21 | CGC_TGC | R | C | TRUE |
| 21 | CGC_CTC | R | L | TRUE |
| 21 | CGC_AGC | R | S | TRUE |
| 21 | CCA_CGA | P | R | TRUE |
| 21 | AAT_ATT | N | I | TRUE |
| 21 | AAC_ATC | N | I | TRUE |
| 21 | AAA_ACA | K | T | TRUE |
| 21 | AAA_AAT | K | N | TRUE |
| 18 | TTA_TTC | L | F | TRUE |
| 18 | TGG_TGC | W | C | TRUE |
| 18 | TGC_AGC | C | S | TRUE |
| 18 | TAT_TCT | Y | S | TRUE |
| 18 | GGG_GTG | G | V | TRUE |
| 18 | GGG_GAG | G | E | TRUE |
| 18 | GGC_GCC | G | A | TRUE |
| 18 | GCT_GAT | A | D | TRUE |
| 18 | GCT_CCT | A | P | TRUE |

| Number of occurrences | Codon change | Amino acid change | | Non-synonymous |
|---|---|---|---|---|
| 18 | GCG_GAG | A | E | TRUE |
| 18 | GAT_GAA | D | E | TRUE |
| 18 | CGT_AGT | R | S | TRUE |
| 18 | CCT_CAT | P | H | TRUE |
| 18 | CCC_CTC | P | L | TRUE |
| 18 | ATG_CTG | M | L | TRUE |
| 18 | AGG_AGT | R | S | TRUE |
| 18 | AGC_AGG | S | R | TRUE |
| 18 | ACT_TCT | T | S | TRUE |
| 18 | AAT_AAG | N | K | TRUE |
| 15 | TTC_TTG | F | L | TRUE |
| 15 | TTC_TTA | F | L | TRUE |
| 15 | TGG_TGT | W | C | TRUE |
| 15 | TCG_CCG | S | P | TRUE |
| 15 | TCC_TAC | S | Y | TRUE |
| 15 | GTT_CTT | V | L | TRUE |
| 15 | GCG_TCG | A | S | TRUE |
| 15 | GAA_GAT | E | D | TRUE |
| 15 | CTT_CCT | L | P | TRUE |
| 15 | CTT_CAT | L | H | TRUE |
| 15 | CTA_CCA | L | P | TRUE |
| 15 | CAA_CAT | Q | H | TRUE |
| 15 | ATC_CTC | I | L | TRUE |
| 15 | AGA_GGA | R | G | TRUE |
| 15 | ACG_AAG | T | K | TRUE |
| 15 | ACC_TCC | T | S | TRUE |
| 15 | AAC_AAA | N | K | TRUE |
| 12 | TGG_TCG | W | S | TRUE |
| 12 | TCT_TGT | S | C | TRUE |
| 12 | TCG_GCG | S | A | TRUE |
| 12 | TAT_TTT | Y | F | TRUE |
| 12 | GGT_GTT | G | V | TRUE |
| 12 | GCA_GAA | A | E | TRUE |
| 12 | GAT_GCT | D | A | TRUE |
| 12 | GAA_GAC | E | D | TRUE |
| 12 | CTG_GTG | L | V | TRUE |
| 12 | CTA_GTA | L | V | TRUE |
| 12 | CTA_ATA | L | I | TRUE |
| 12 | CGC_CAC | R | H | TRUE |
| 12 | ATC_TTC | I | F | TRUE |
| 12 | ATC_ATG | I | M | TRUE |
| 12 | AGT_CGT | S | R | TRUE |
| 12 | AGT_AGG | S | R | TRUE |
| 12 | AGC_ATC | S | I | TRUE |
| 12 | AGA_AGT | R | S | TRUE |
| 12 | AGA_AGC | R | S | TRUE |
| 12 | ACC_CCC | T | P | TRUE |
| 12 | AAT_ACT | N | T | TRUE |
| 12 | AAG_CAG | K | Q | TRUE |
| 9 | TTT_TCT | F | S | TRUE |
| 9 | TTG_TTT | L | F | TRUE |

| Number of occurrences | Codon change | Amino acid change | | Non-synonymous |
|---|---|---|---|---|
| 9 | TGT_TGG | C | W | TRUE |
| 9 | TGG_TTG | W | L | TRUE |
| 9 | TGC_TCC | C | S | TRUE |
| 9 | TGC_TAC | C | Y | TRUE |
| 9 | TAT_AAT | Y | N | TRUE |
| 9 | GTG_TTG | V | L | TRUE |
| 9 | GTC_TTC | V | F | TRUE |
| 9 | GTA_GTG | Y | C | TRUE |
| 9 | GTA_CTA | V | L | TRUE |
| 9 | GGC_GAC | G | D | TRUE |
| 9 | GGA_GTA | G | V | TRUE |
| 9 | GCC_GAC | A | D | TRUE |
| 9 | GAT_GTT | D | V | TRUE |
| 9 | GAG_GGG | E | G | TRUE |
| 9 | GAA_GCA | E | A | TRUE |
| 9 | CTT_GTT | L | V | TRUE |
| 9 | CTC_CCC | L | P | TRUE |
| 9 | CCG_TCG | P | S | TRUE |
| 9 | CCC_GCC | P | A | TRUE |
| 9 | CAT_CAG | H | Q | TRUE |
| 9 | CAG_AAG | Q | K | TRUE |
| 9 | ATT_AGT | I | S | TRUE |
| 9 | ATG_ATC | M | I | TRUE |
| 9 | ACA_TCA | T | S | TRUE |
| 9 | AAA_AAC | K | N | TRUE |
| 6 | TTT_TTG | F | L | TRUE |
| 6 | TTG_TTC | L | F | TRUE |
| 6 | TTC_TGC | F | C | TRUE |
| 6 | TTC_GTC | F | V | TRUE |
| 6 | TGG_GGG | W | G | TRUE |
| 6 | TCA_GCA | S | A | TRUE |
| 6 | GTT_GAT | V | D | TRUE |
| 6 | GTG_GAG | V | E | TRUE |
| 6 | GTA_GCA | V | A | TRUE |
| 6 | GTA_GAA | V | E | TRUE |
| 6 | GGT_TGT | G | C | TRUE |
| 6 | GGG_TGG | G | W | TRUE |
| 6 | GGG_GCG | G | A | TRUE |
| 6 | GGA_GCA | G | A | TRUE |
| 6 | GAT_TAT | D | Y | TRUE |
| 6 | GAT_CAT | D | H | TRUE |
| 6 | CTC_GTC | L | V | TRUE |
| 6 | CGG_CCG | R | P | TRUE |
| 6 | CGC_CCC | R | P | TRUE |
| 6 | CCA_GCA | P | A | TRUE |
| 6 | CCA_ACA | P | T | TRUE |
| 6 | CAT_CAA | H | Q | TRUE |
| 6 | CAT_AAT | H | N | TRUE |
| 6 | CAG_CTG | Q | L | TRUE |
| 6 | CAG_CAC | Q | H | TRUE |
| 6 | CAC_GAC | H | D | TRUE |

| Number of occurrences | Codon change | Amino acid change | | Non-synonymous |
|---|---|---|---|---|
| 6 | ATG_AAG | M | K | TRUE |
| 6 | ATA_CTA | I | L | TRUE |
| 6 | ATA_AAA | I | K | TRUE |
| 6 | AGT_ATT | S | I | TRUE |
| 6 | AGG_ATG | R | M | TRUE |
| 6 | ACG_GCG | T | A | TRUE |
| 6 | AAT_CAT | N | H | TRUE |
| 6 | AAG_ACG | K | T | TRUE |
| 6 | AAC_CAC | N | H | TRUE |
| 3 | TGT_TTT | C | F | TRUE |
| 3 | TGT_AGT | C | S | TRUE |
| 3 | TGC_TGG | C | W | TRUE |
| 3 | TCT_TAT | S | Y | TRUE |
| 3 | TAC_TGC | Y | C | TRUE |
| 3 | N/A_N/A | T | M | TRUE |
| 3 | N/A_N/A | S | T | TRUE |
| 3 | N/A_N/A | R | Q | TRUE |
| 3 | GTG_GGG | V | G | TRUE |
| 3 | GTC_GGC | V | G | TRUE |
| 3 | GTA_TTA | V | L | TRUE |
| 3 | GGT_GCT | G | A | TRUE |
| 3 | GGG_CGG | G | R | TRUE |
| 3 | GGC_GTC | G | V | TRUE |
| 3 | GGA_CGA | G | R | TRUE |
| 3 | GCG_GGG | A | G | TRUE |
| 3 | GCG_CCG | A | P | TRUE |
| 3 | GAC_GTC | D | V | TRUE |
| 3 | GAC_GCC | D | A | TRUE |
| 3 | CTG_CGG | L | R | TRUE |
| 3 | CTC_CAC | L | H | TRUE |
| 3 | CGT_GGT | R | G | TRUE |
| 3 | CGT_CCT | R | P | TRUE |
| 3 | CGC_GGC | R | G | TRUE |
| 3 | CGA_GGA | R | G | TRUE |
| 3 | CCT_ACT | P | T | TRUE |
| 3 | CCG_CAG | P | Q | TRUE |
| 3 | CCG_ACG | P | T | TRUE |
| 3 | CCC_CGC | P | R | TRUE |
| 3 | CCA_CAA | P | Q | TRUE |
| 3 | CAT_GAT | H | D | TRUE |
| 3 | CAC_CTC | H | L | TRUE |
| 3 | CAC_CAT | V | M | TRUE |
| 3 | CAC_CAA | H | Q | TRUE |
| 3 | CAA_CTA | Q | L | TRUE |
| 3 | ATT_TTT | I | F | TRUE |
| 3 | ATT_AAT | I | N | TRUE |
| 3 | ATA_TTA | I | L | TRUE |
| 3 | ATA_AGA | I | R | TRUE |
| 3 | AGC_CGC | S | R | TRUE |
| 3 | AGA_ATA | R | I | TRUE |
| 3 | ACT_CCT | T | P | TRUE |

| Number of occurrences | Codon change | Amino acid change | | Non-synonymous |
|---|---|---|---|---|
| 3 | ACT_AAT | T | N | TRUE |
| 3 | ACA_AAA | T | K | TRUE |
| 3 | AAT_AAA | N | K | TRUE |
| 3 | AAG_ATG | K | M | TRUE |

**Table S5.** Brain synonymous/non-synonymous amino acid substitution.

| Number of occurrences | Codon change | Amino acid change | | Non-synonymous |
|---|---|---|---|---|
| 105 | GAC_GAT | D | D | FALSE |
| 85 | CCA_CCG | P | P | FALSE |
| 80 | CCG_CCA | P | P | FALSE |
| 78 | GAA_GAG | E | E | FALSE |
| 75 | GCA_GCG | A | A | FALSE |
| 75 | GAG_GAA | E | E | FALSE |
| 74 | ACG_ACA | T | T | FALSE |
| 71 | GCT_GCC | A | A | FALSE |
| 71 | AAC_AAT | N | N | FALSE |
| 69 | ATT_ATC | I | I | FALSE |
| 68 | GCC_GCT | A | A | FALSE |
| 68 | GAT_GAC | D | D | FALSE |
| 67 | TAC_TAT | Y | Y | FALSE |
| 63 | CAG_CAA | Q | Q | FALSE |
| 60 | ACA_ACG | T | T | FALSE |
| 60 | AAT_AAC | N | N | FALSE |
| 59 | CAC_CAT | H | H | FALSE |
| 59 | CAA_CAG | Q | Q | FALSE |
| 57 | AAA_AAG | K | K | FALSE |
| 56 | ATC_ATT | I | I | FALSE |
| 52 | TCA_TCG | S | S | FALSE |
| 50 | CTG_TTG | L | L | FALSE |
| 47 | GGT_GGC | G | G | FALSE |
| 46 | AGC_AGT | S | S | FALSE |
| 45 | TAT_TAC | Y | Y | FALSE |
| 45 | GTA_GTG | V | V | FALSE |
| 44 | CTT_CTC | L | L | FALSE |
| 44 | AGT_AGC | S | S | FALSE |
| 44 | ACC_ACT | T | T | FALSE |
| 43 | CAT_CAC | H | H | FALSE |
| 41 | TCC_TCT | S | S | FALSE |
| 41 | CCT_CCC | P | P | FALSE |
| 39 | GTT_GTC | V | V | FALSE |
| 37 | GCG_GCA | A | A | FALSE |
| 36 | CTG_CTA | L | L | FALSE |
| 36 | CCC_CCT | P | P | FALSE |
| 34 | TTT_TTC | F | F | FALSE |
| 34 | ACT_ACC | T | T | FALSE |
| 33 | TTG_CTG | L | L | FALSE |
| 33 | GGG_GGA | G | G | FALSE |
| 33 | CTA_CTG | L | L | FALSE |
| 32 | GGC_GGT | G | G | FALSE |
| 31 | TGC_TGT | C | C | FALSE |
| 31 | GGA_GGG | G | G | FALSE |

| Number of occurrences | Codon change | Amino acid change | | Non-synonymous |
|---|---|---|---|---|
| 28 | GTC_GTT | V | V | FALSE |
| 28 | CTC_CTT | L | L | FALSE |
| 27 | GTG_GTA | V | V | FALSE |
| 27 | CGC_CGT | R | R | FALSE |
| 25 | CTG_CTT | L | L | FALSE |
| 24 | TCG_TCA | S | S | FALSE |
| 24 | AAG_AAA | K | K | FALSE |
| 23 | TTG_TTA | L | L | FALSE |
| 23 | AGA_AGG | R | R | FALSE |
| 22 | TGT_TGC | C | C | FALSE |
| 22 | TCC_TCG | S | S | FALSE |
| 20 | TTC_TTT | F | F | FALSE |
| 19 | TCT_TCC | S | S | FALSE |
| 19 | GTG_GTT | V | V | FALSE |
| 19 | CTC_CTG | L | L | FALSE |
| 19 | CTA_TTA | L | L | FALSE |
| 18 | GCA_GCT | A | A | FALSE |
| 18 | CGA_CGG | R | R | FALSE |
| 18 | CCG_CCT | P | P | FALSE |
| 17 | CGT_CGC | R | R | FALSE |
| 17 | AGG_AGA | R | R | FALSE |
| 15 | TCA_TCT | S | S | FALSE |
| 15 | CGA_AGA | R | R | FALSE |
| 15 | CCA_CCT | P | P | FALSE |
| 14 | ATC_ATA | I | I | FALSE |
| 14 | ACC_ACA | T | T | FALSE |
| 13 | GGC_GGG | G | G | FALSE |
| 13 | CTC_CTA | L | L | FALSE |
| 13 | CCC_CCA | P | P | FALSE |
| 12 | CTG_CTC | L | L | FALSE |
| 11 | GTC_GTG | V | V | FALSE |
| 11 | GGA_GGC | G | G | FALSE |
| 11 | CGG_CGA | R | R | FALSE |
| 11 | CGG_AGG | R | R | FALSE |
| 10 | TTA_CTA | L | L | FALSE |
| 10 | GTC_GTA | V | V | FALSE |
| 10 | ATA_ATT | I | I | FALSE |
| 10 | ACG_ACT | T | T | FALSE |
| 8 | TTA_TTG | L | L | FALSE |
| 8 | GTG_GTC | V | V | FALSE |
| 8 | GCT_GCA | A | A | FALSE |
| 8 | CCA_CCC | P | P | FALSE |
| 7 | TCG_TCC | S | S | FALSE |
| 7 | GGG_GGT | G | G | FALSE |
| 7 | GGA_GGT | G | G | FALSE |
| 7 | GCG_GCT | A | A | FALSE |
| 7 | GCC_GCA | A | A | FALSE |
| 7 | CTT_CTA | L | L | FALSE |
| 7 | ATT_ATA | I | I | FALSE |
| 6 | TCG_TCT | S | S | FALSE |
| 6 | TCC_TCA | S | S | FALSE |

| Number of occurrences | Codon change | Amino acid change | | Non-synonymous |
|---|---|---|---|---|
| 6 | GTT_GTA | V | V | FALSE |
| 6 | GGC_GGA | G | G | FALSE |
| 6 | CTA_CTC | L | L | FALSE |
| 6 | CGC_CGA | R | R | FALSE |
| 6 | AGA_CGA | R | R | FALSE |
| 6 | ACT_ACA | T | T | FALSE |
| 6 | ACA_ACT | T | T | FALSE |
| 5 | GCG_GCC | A | A | FALSE |
| 5 | GCC_GCG | A | A | FALSE |
| 5 | CCT_CCA | P | P | FALSE |
| 4 | TCT_TCG | S | S | FALSE |
| 4 | N/A_N/A | A | A | FALSE |
| 4 | GGG_GGC | G | G | FALSE |
| 4 | GCA_GCC | A | A | FALSE |
| 4 | CGA_CGT | R | R | FALSE |
| 4 | CCG_CCC | P | P | FALSE |
| 4 | ATA_ATC | I | I | FALSE |
| 3 | TCT_TCA | S | S | FALSE |
| 3 | N/A_N/A | V | V | FALSE |
| 3 | GTA_GTT | V | V | FALSE |
| 3 | CTA_CTT | L | L | FALSE |
| 3 | CGG_CGT | R | R | FALSE |
| 3 | ACC_ACG | T | T | FALSE |
| 2 | N/A_N/A | P | P | FALSE |
| 2 | N/A_N/A | L | L | FALSE |
| 2 | GGA_AGA | R | R | FALSE |
| 2 | GCT_GCG | A | A | FALSE |
| 2 | CTT_CTG | L | L | FALSE |
| 2 | ACG_ACC | T | T | FALSE |
| 2 | AAA_AGA | E | E | FALSE |
| 1 | TCA_TCC | S | S | FALSE |
| 1 | N/A_N/A | Q | Q | FALSE |
| 1 | GTT_GTG | V | V | FALSE |
| 1 | GTA_GTC | V | V | FALSE |
| 1 | GGT_GGG | G | G | FALSE |
| 1 | GGT_GGA | G | G | FALSE |
| 1 | CTA_CAA | T | T | FALSE |
| 1 | CGG_CGC | R | R | FALSE |
| 1 | CGC_CGG | R | R | FALSE |
| 1 | CCT_CCG | P | P | FALSE |
| 1 | CCC_CCG | P | P | FALSE |
| 1 | AGG_CGG | R | R | FALSE |
| 1 | ACG_GCG | E | E | FALSE |
| 1 | AAG_GAG | E | E | FALSE |
| 22 | GTG_CTG | V | L | TRUE |
| 22 | AAA_AGA | K | R | TRUE |
| 19 | AGT_AAT | S | N | TRUE |
| 18 | GAG_CAG | E | Q | TRUE |
| 18 | AAC_GAC | N | D | TRUE |
| 17 | GTG_ATG | V | M | TRUE |
| 17 | GGT_AGT | G | S | TRUE |

| Number of occurrences | Codon change | Amino acid change | | Non-synonymous |
|---|---|---|---|---|
| 17 | CAG_GAG | Q | E | TRUE |
| 17 | ATT_ACT | I | T | TRUE |
| 16 | TTC_CTC | F | L | TRUE |
| 16 | GTG_GCG | V | A | TRUE |
| 16 | AAG_GAG | K | E | TRUE |
| 15 | GAA_GGA | E | G | TRUE |
| 15 | CGA_CAA | R | Q | TRUE |
| 15 | CAA_CGA | Q | R | TRUE |
| 15 | AGA_AAA | R | K | TRUE |
| 14 | TTT_TCT | F | S | TRUE |
| 14 | GTA_ATA | V | I | TRUE |
| 14 | GAC_GAG | D | E | TRUE |
| 14 | CCT_TCT | P | S | TRUE |
| 14 | ATG_GTG | M | V | TRUE |
| 13 | GTT_ATT | V | I | TRUE |
| 13 | GCT_ACT | A | T | TRUE |
| 13 | CGC_CAC | R | H | TRUE |
| 13 | AGG_GGG | R | G | TRUE |
| 13 | ACG_ATG | T | M | TRUE |
| 12 | TCT_TTT | S | F | TRUE |
| 12 | GGA_GAA | G | E | TRUE |
| 12 | AGA_GGA | R | G | TRUE |
| 12 | AGA_ACA | R | T | TRUE |
| 12 | ACA_ATA | T | I | TRUE |
| 11 | TGT_TCT | C | S | TRUE |
| 11 | TGT_TAT | C | Y | TRUE |
| 11 | TCC_ACC | S | T | TRUE |
| 11 | GAG_GGG | E | G | TRUE |
| 11 | ATC_GTC | I | V | TRUE |
| 10 | GTC_GAC | V | D | TRUE |
| 10 | ATA_ACA | I | T | TRUE |
| 10 | ACT_GCT | T | A | TRUE |
| 10 | AAT_AGT | N | S | TRUE |
| 9 | GTC_ATC | V | I | TRUE |
| 9 | GCA_ACA | A | T | TRUE |
| 9 | GAC_TAC | D | Y | TRUE |
| 9 | CTT_CCT | L | P | TRUE |
| 9 | CCT_GCT | P | A | TRUE |
| 9 | CCG_CTG | P | L | TRUE |
| 9 | CAC_CGC | H | R | TRUE |
| 9 | ATA_ATG | I | M | TRUE |
| 9 | AGC_AAC | S | N | TRUE |
| 9 | ACC_AAC | T | N | TRUE |
| 9 | ACA_AGA | T | R | TRUE |
| 9 | AAA_GAA | K | E | TRUE |
| 8 | TGC_TCC | C | S | TRUE |
| 8 | TCT_CCT | S | P | TRUE |
| 8 | TAT_TGT | Y | C | TRUE |
| 8 | GTC_CTC | V | L | TRUE |
| 8 | GGG_AGG | G | R | TRUE |
| 8 | GCC_ACC | A | T | TRUE |

| Number of occurrences | Codon change | Amino acid change | | Non-synonymous |
|---|---|---|---|---|
| 8 | GAG_AAG | E | K | TRUE |
| 8 | CTC_TTC | L | F | TRUE |
| 8 | CCC_TCC | P | S | TRUE |
| 8 | CCA_CAA | P | Q | TRUE |
| 8 | CAG_CAC | Q | H | TRUE |
| 8 | ATT_GTT | I | V | TRUE |
| 8 | ACC_GCC | T | A | TRUE |
| 8 | AAG_AGG | K | R | TRUE |
| 7 | TTC_TCC | F | S | TRUE |
| 7 | CAT_TAT | H | Y | TRUE |
| 7 | CAA_CTA | Q | L | TRUE |
| 7 | ATG_ACG | M | T | TRUE |
| 7 | ATA_AGA | I | R | TRUE |
| 7 | AAC_AGC | N | S | TRUE |
| 6 | TCT_TGT | S | C | TRUE |
| 6 | TCA_CCA | S | P | TRUE |
| 6 | TAT_CAT | Y | H | TRUE |
| 6 | GTT_CTT | V | L | TRUE |
| 6 | GGA_AGA | G | R | TRUE |
| 6 | GCG_GTG | A | V | TRUE |
| 6 | GCC_GTC | A | V | TRUE |
| 6 | CTC_ATC | L | I | TRUE |
| 6 | CCC_CTC | P | L | TRUE |
| 6 | CAG_AAG | Q | K | TRUE |
| 6 | CAC_CAG | H | Q | TRUE |
| 5 | TTA_ATA | L | I | TRUE |
| 5 | TCT_TAT | S | Y | TRUE |
| 5 | TCC_TTC | S | F | TRUE |
| 5 | GGC_GAC | G | D | TRUE |
| 5 | GCT_CCT | A | P | TRUE |
| 5 | GAT_GTT | D | V | TRUE |
| 5 | GAT_GCT | D | A | TRUE |
| 5 | GAT_GAG | D | E | TRUE |
| 5 | GAG_GAT | E | D | TRUE |
| 5 | GAA_GAT | E | D | TRUE |
| 5 | GAA_CAA | E | Q | TRUE |
| 5 | CTT_ATT | L | I | TRUE |
| 5 | CTC_GTC | L | V | TRUE |
| 5 | CTA_CCA | L | P | TRUE |
| 5 | CGA_CTA | R | L | TRUE |
| 5 | CCT_ACT | P | T | TRUE |
| 5 | CCA_CTA | P | L | TRUE |
| 5 | CAC_CAA | H | Q | TRUE |
| 5 | ATC_ACC | I | T | TRUE |
| 5 | AGT_GGT | S | G | TRUE |
| 5 | AGT_AGG | S | R | TRUE |
| 5 | AGG_AGT | R | S | TRUE |
| 5 | ACT_TCT | T | S | TRUE |
| 5 | ACT_ATT | T | I | TRUE |
| 5 | ACT_AGT | T | S | TRUE |
| 4 | TTG_TTT | L | F | TRUE |

| Number of occurrences | Codon change | Amino acid change | | Non-synonymous |
|---|---|---|---|---|
| 4 | TTG_TCG | L | S | TRUE |
| 4 | TTG_ATG | L | M | TRUE |
| 4 | GTT_GCT | V | A | TRUE |
| 4 | GTC_TTC | V | F | TRUE |
| 4 | GTC_GCC | V | A | TRUE |
| 4 | GTA_TTA | V | L | TRUE |
| 4 | GTA_GCA | V | A | TRUE |
| 4 | GGG_GAG | G | E | TRUE |
| 4 | GCT_TCT | A | S | TRUE |
| 4 | CTT_TTT | L | F | TRUE |
| 4 | CTG_GTG | L | V | TRUE |
| 4 | CTC_CCC | L | P | TRUE |
| 4 | CGC_AGC | R | S | TRUE |
| 4 | CAG_CGG | Q | R | TRUE |
| 4 | ATG_ATA | M | I | TRUE |
| 4 | AGT_ACT | S | T | TRUE |
| 4 | AGG_AGC | R | S | TRUE |
| 4 | AGG_AAG | R | K | TRUE |
| 4 | AAT_GAT | N | D | TRUE |
| 4 | AAA_ATA | K | I | TRUE |
| 3 | TTT_CTT | F | L | TRUE |
| 3 | TTC_TTG | F | L | TRUE |
| 3 | TTC_GTC | F | V | TRUE |
| 3 | TGT_TTT | C | F | TRUE |
| 3 | TCC_CCC | S | P | TRUE |
| 3 | N/A_N/A | R | H | TRUE |
| 3 | N/A_N/A | K | E | TRUE |
| 3 | GTT_TTT | V | F | TRUE |
| 3 | GTG_GGG | V | G | TRUE |
| 3 | GCA_TCA | A | S | TRUE |
| 3 | GCA_GAA | A | E | TRUE |
| 3 | GAG_GAC | E | D | TRUE |
| 3 | GAC_AAC | D | N | TRUE |
| 3 | CTG_ATG | L | M | TRUE |
| 3 | CTA_GTA | L | V | TRUE |
| 3 | CGT_TGT | R | C | TRUE |
| 3 | CGG_TGG | R | W | TRUE |
| 3 | CGG_CAG | R | Q | TRUE |
| 3 | CCA_ACA | P | T | TRUE |
| 3 | CAT_AAT | H | N | TRUE |
| 3 | CAG_CAT | Q | H | TRUE |
| 3 | CAC_TAC | H | Y | TRUE |
| 3 | CAA_GAA | Q | E | TRUE |
| 3 | CAA_CAT | Q | H | TRUE |
| 3 | ATG_ATT | M | I | TRUE |
| 3 | ATG_ATC | M | I | TRUE |
| 3 | ATC_TTC | I | F | TRUE |
| 3 | ATA_AAA | I | K | TRUE |
| 3 | AGT_AGA | S | R | TRUE |
| 3 | AGC_AGG | S | R | TRUE |
| 3 | AGC_AGA | S | R | TRUE |

| Number of occurrences | Codon change | Amino acid change | | Non-synonymous |
|---|---|---|---|---|
| 3 | ACG_AAG | T | K | TRUE |
| 3 | ACC_ATC | T | I | TRUE |
| 3 | ACA_GCA | T | A | TRUE |
| 2 | TTT_TTG | F | L | TRUE |
| 2 | TTG_TTC | L | F | TRUE |
| 2 | TTG_GTG | L | V | TRUE |
| 2 | TTA_TTT | L | F | TRUE |
| 2 | TGT_AGT | C | S | TRUE |
| 2 | TGG_TGC | W | C | TRUE |
| 2 | TGC_CGC | C | R | TRUE |
| 2 | TCT_ACT | S | T | TRUE |
| 2 | TAC_TGC | Y | C | TRUE |
| 2 | N/A_N/A | V | I | TRUE |
| 2 | N/A_N/A | Q | P | TRUE |
| 2 | GTG_TTG | V | L | TRUE |
| 2 | GGT_GTT | G | V | TRUE |
| 2 | GGT_GAT | G | D | TRUE |
| 2 | GGC_AGC | G | S | TRUE |
| 2 | GGA_GTA | G | V | TRUE |
| 2 | GGA_CGA | G | R | TRUE |
| 2 | GCT_GAT | A | D | TRUE |
| 2 | GCG_ACG | A | T | TRUE |
| 2 | GCA_GGA | A | G | TRUE |
| 2 | GAT_TAT | D | Y | TRUE |
| 2 | GAT_GGT | D | G | TRUE |
| 2 | GAT_CAT | D | H | TRUE |
| 2 | GAT_AAT | D | N | TRUE |
| 2 | GAG_GAT | D | Y | TRUE |
| 2 | GAC_GTC | D | V | TRUE |
| 2 | GAC_GGC | D | G | TRUE |
| 2 | GAA_AAA | E | K | TRUE |
| 2 | CTT_GTT | L | V | TRUE |
| 2 | CTT_CAT | L | H | TRUE |
| 2 | CTG_CCG | L | P | TRUE |
| 2 | CTG_CAG | L | Q | TRUE |
| 2 | CTC_CGC | L | R | TRUE |
| 2 | CTC_CAC | L | H | TRUE |
| 2 | CTA_CAA | L | Q | TRUE |
| 2 | CGC_TGC | R | C | TRUE |
| 2 | CGC_CTC | R | L | TRUE |
| 2 | CCT_CTT | P | L | TRUE |
| 2 | CCC_CGC | P | R | TRUE |
| 2 | CCA_TCA | P | S | TRUE |
| 2 | CCA_GCA | P | A | TRUE |
| 2 | CAG_TAG | T | I | TRUE |
| 2 | CAC_GAC | H | D | TRUE |
| 2 | CAC_CTC | H | L | TRUE |
| 2 | ATT_TTT | I | F | TRUE |
| 2 | ATG_AAG | M | K | TRUE |
| 2 | ATA_GTA | I | V | TRUE |
| 2 | AGT_ATT | S | I | TRUE |

| Number of occurrences | Codon change | Amino acid change | | Non-synonymous |
|---|---|---|---|---|
| 2 | AGA_ATA | R | I | TRUE |
| 2 | ACT_CCT | T | P | TRUE |
| 2 | ACG_GCG | T | A | TRUE |
| 2 | ACC_TCC | T | S | TRUE |
| 2 | AAT_TAT | N | Y | TRUE |
| 2 | AAC_ATC | N | I | TRUE |
| 2 | AAC_ACC | N | T | TRUE |
| 1 | TTT_TTA | F | L | TRUE |
| 1 | TTT_TGT | F | C | TRUE |
| 1 | TTT_GTT | F | V | TRUE |
| 1 | TTG_TGG | L | W | TRUE |
| 1 | TTC_TAC | F | Y | TRUE |
| 1 | TTA_TCA | L | S | TRUE |
| 1 | TTA_GTA | L | V | TRUE |
| 1 | TGT_CGT | C | R | TRUE |
| 1 | TGG_TGT | W | C | TRUE |
| 1 | TGG_CGG | W | R | TRUE |
| 1 | TGC_TTC | C | F | TRUE |
| 1 | TGC_AGC | C | S | TRUE |
| 1 | TCT_GCT | S | A | TRUE |
| 1 | TCG_TTG | S | L | TRUE |
| 1 | TCG_TGG | S | W | TRUE |
| 1 | TCC_TGC | S | C | TRUE |
| 1 | TCC_TAC | S | Y | TRUE |
| 1 | TAT_TTT | Y | F | TRUE |
| 1 | TAC_TCC | Y | S | TRUE |
| 1 | TAC_CAC | Y | H | TRUE |
| 1 | N/A_N/A | I | V | TRUE |
| 1 | N/A_N/A | F | S | TRUE |
| 1 | N/A_N/A | C | Y | TRUE |
| 1 | GTG_GAG | V | E | TRUE |
| 1 | GTG_GAG | S | R | TRUE |
| 1 | GTG_CTG | S | T | TRUE |
| 1 | GTA_GAA | V | E | TRUE |
| 1 | GTA_CTA | V | L | TRUE |
| 1 | GGT_CGT | G | R | TRUE |
| 1 | GGG_GTG | G | V | TRUE |
| 1 | GGG_GCG | G | A | TRUE |
| 1 | GGG_CGG | G | R | TRUE |
| 1 | GGC_GTC | R | S | TRUE |
| 1 | GGC_GTC | G | V | TRUE |
| 1 | GGC_GCC | G | A | TRUE |
| 1 | GGA_GTA | D | Y | TRUE |
| 1 | GGA_GGG | E | G | TRUE |
| 1 | GGA_CGA | G | A | TRUE |
| 1 | GCC_TCC | A | S | TRUE |
| 1 | GCC_CCC | A | P | TRUE |
| 1 | GCA_GTA | A | V | TRUE |
| 1 | GAG_GGG | R | G | TRUE |
| 1 | GAG_GCG | E | A | TRUE |
| 1 | GAG_GAC | E | Q | TRUE |

| Number of occurrences | Codon change | Amino acid change | | Non-synonymous |
|---|---|---|---|---|
| 1 | GAC_GAG | Q | E | TRUE |
| 1 | GAA_GTA | E | V | TRUE |
| 1 | GAA_GCA | E | A | TRUE |
| 1 | CTT_CGT | L | R | TRUE |
| 1 | CTA_ATA | L | I | TRUE |
| 1 | CGT_CCT | R | P | TRUE |
| 1 | CGT_CAT | R | H | TRUE |
| 1 | CGG_GGG | R | G | TRUE |
| 1 | CCT_CAT | P | H | TRUE |
| 1 | CCG_TCG | P | S | TRUE |
| 1 | CCG_GCG | P | A | TRUE |
| 1 | CCG_CAG | P | Q | TRUE |
| 1 | CCC_ACC | P | T | TRUE |
| 1 | CCA_CGA | P | R | TRUE |
| 1 | CAT_CTT | H | L | TRUE |
| 1 | CAT_CGT | H | R | TRUE |
| 1 | CAT_CCT | H | P | TRUE |
| 1 | CAT_CAA | H | Q | TRUE |
| 1 | CAG_CTG | Q | L | TRUE |
| 1 | CAG_CCG | Q | P | TRUE |
| 1 | CAG_CAC | E | Q | TRUE |
| 1 | CAC_CAG | Q | E | TRUE |
| 1 | CAA_CAC | Q | H | TRUE |
| 1 | ATG_TTG | M | L | TRUE |
| 1 | ATC_CTC | I | L | TRUE |
| 1 | ATC_AGC | I | S | TRUE |
| 1 | ATA_TTA | I | L | TRUE |
| 1 | ATA_CTA | I | L | TRUE |
| 1 | AGT_AGA | C | S | TRUE |
| 1 | AGG_CGG | E | A | TRUE |
| 1 | AGG_ATG | R | M | TRUE |
| 1 | AGG_AGC | E | Q | TRUE |
| 1 | AGC_GGC | S | G | TRUE |
| 1 | AGC_AGA | P | T | TRUE |
| 1 | AGA_AGC | R | S | TRUE |
| 1 | ACC_CCC | T | P | TRUE |
| 1 | ACA_TCA | T | S | TRUE |
| 1 | ACA_AGA | Q | E | TRUE |
| 1 | AAT_AAA | N | K | TRUE |
| 1 | AAG_AAT | K | N | TRUE |
| 1 | AAC_CAC | N | H | TRUE |
| 1 | AAC_AAG | N | K | TRUE |
| 1 | AAA_CAA | K | Q | TRUE |
| 1 | AAA_AAG | R | G | TRUE |
| 1 | AAA_AAC | K | N | TRUE |
